# Distinct *Arnica montana* L. extracts modulate human T cell activation in different ways via differential inhibition of NFκB and NFAT pathways

**DOI:** 10.3389/fimmu.2025.1655212

**Published:** 2025-10-15

**Authors:** Karina M. Berschneider, Bernhard Wetterauer, Carsten Sticht, Christian Orlik, Beate Jahraus, Henning Kirchgessner, Anastasiia Zuieva, Stefan Wölfl, Divya Lairikyengbam, Verena Beier, Guido Wabnitz, Pille Wetterauer, Michael Schmiech, Yvonne Samstag

**Affiliations:** 1 Section Molecular Immunology, Institute of Immunology, Heidelberg University Hospital, Heidelberg, Germany; 2 Institute of Pharmacy and Molecular Biotechnology, Heidelberg University, Heidelberg, Germany; 3 Next Generation Sequencing Core Facility, Medical Faculty Mannheim, Heidelberg University, Mannheim, Germany; 4 Institute of Experimental and Clinical Pharmacology, Toxicology and Pharmacology of Natural Products, University of Ulm, Ulm, Germany

**Keywords:** *Arnica montana* L. extracts, primary human T cells (PBTs), immunomodulation, NFκB, NFAT, thymol, helenalin, LC-MS/MS

## Abstract

*Arnica montana* L. (Arnica) has a long history of use in treating inflammation and soft tissue injury, yet its immunomodulatory mechanisms remain largely unexplored. In this study, we investigated the effects of distinct Arnica extracts - derived from different plant parts (root or whole plant) and manufacturing processes - on primary human T cells. We also compared their effects with those of the pure compounds helenalin and thymol. All extracts inhibited T cell activation and proliferation. This could be traced back to reduced IL-2 responsiveness due to decreased CD25 (IL–2Rα chain) expression, accompanied by reduced IL-2 production. Transcriptomic analysis (nCounter) and gene set enrichment revealed that the extracts target key T cell receptor (TCR) signaling pathways. Mechanistically, the hydroethanolic root extract selectively inhibited NFκB DNA binding, while the aqueous fermented extract predominantly suppressed NFAT-dependent gene expression. The hydroethanolic whole plant extract exerted a moderate effect on both pathways. These findings identify Arnica extracts as promising modulators of human TCR signaling and support their potential in regulating T cell-driven inflammatory responses, with implications for muscle healing and chronic inflammatory diseases.

## 1 Introduction

For centuries, *Arnica montana* L. (Arnica) preparations have been used to treat inflammation and to promote the healing of blunt injuries, such as contusions and bruises (1). A total of about 150 therapeutically active substances have been identified in the different plant parts of Arnica (1). Its anti-inflammatory mode of action has been mainly attributed to the contained sesquiterpene lactones (SLs), with helenalin as the most prominent representative (2–4). Nuclear factor ‘kappa-light-chain-enhancer’ of activated B cells (NFκB) regulates the expression of a variety of immunity- and inflammation-related genes and was identified as molecular target of helenalin in the T leukemia line Jurkat (5). Helenalin is mainly contained in the flower heads (6, 7), whereas thymol (monoterpene phenol) and its derivatives are enriched in the essential oil of underground plant parts (8, 9). Another study described an inhibitory effect of Arnica flower tincture on nuclear factor of activated T cells (NFAT)-dependent IL-1ß and TNFα release following LPS stimulation of peripheral blood mononuclear cells (2).

The human immune system fulfils the important function of protecting the body from invading pathogens and clearing emerging infections. Apart from that, various immune cell types, including CD4^+^ T cells, are critically involved in the muscle healing process in response to trauma. They invade the injured site in a spatiotemporally controlled manner and rapidly establish a pro-inflammatory milieu that essentially contributes to normal tissue repair (10, 11). CD4^+^ T cells play a key regulatory role and coordinate the complex healing process by means of secreting either pro- or anti-inflammatory cytokines, depending on the T helper subtype. In addition, regulatory T cells promote tissue regeneration by secreting growth factors like amphiregulin (12) and directly interact with activated muscle stem cells to maintain their proliferation and survival prior to differentiation (13). However, prolonged presence of both, CD4^+^ and CD8^+^ T cells, going along with sustained inflammation is a hallmark of delayed muscle repair (10, 14).

Therefore, understanding the effects of Arnica preparations on T cell functions can provide novel insights and mechanistic explanations for critically evaluating their anti-inflammatory effects and their potential for promoting tissue regeneration. Currently, publications investigating the immunomodulatory potential of Arnica extracts are scarce, underscoring the urgent need for more research in this area. Notably, no studies have investigated the effect of Arnica preparations on the activation of primary human T cells upon antigen recognition through the antigen-specific T cell receptor. In order to evaluate potential interactions with conventional immune modulators, the molecular effects of Arnica on human T cells must also be clarified.

T cells require two independent signals to become activated. Under physiological conditions, the first signal is transmitted by ligation of the antigen-specific T cell receptor (TCR) to the respective peptide-MHC complex on antigen-presenting cells (APCs). For the second signal, a costimulatory receptor like CD28 expressed by T cells needs to bind to its ligand CD80 or CD86 on APCs (15). T cell activation induces proliferation and various effector functions including cytokine production. Via their chemokine receptors, T cells are furthermore able to migrate along a chemoattractant gradient. This function allows them to invade sites of infection or injury.

Herein, we characterized the effects of three differentially manufactured Arnica extracts on primary human peripheral blood T cells (PBTs) at a functional and molecular level.

In parallel, the effects of the pure compounds helenalin and thymol on human PBTs were determined. We describe an immunomodulatory potential for each test candidate. Surprisingly, despite similar functional effects, different molecular mechanisms involving the NFκB and/or the NFAT signaling pathway were unraveled. Characterization of the extracts by LC-MS/MS confirmed the assumed variation in phytochemical composition, arising from differences in the used plant part, the extraction solvent and the extraction procedure. These phytochemical differences may explain their different molecular mode of action.

## 2 Materials and methods

### 2.1 Plant extracts and substances

Three differentially prepared Arnica extracts were directly compared in this study. Aqueous fermented extract (WALA Heilmittel GmbH, Bad Boll, Germany) was manufactured according to the production method 33c laid down in the official German Homeopathic Pharmacopoeia (GHP). In short, comminuted fresh flowering plant material (fresh plant material: honey: water: lactose monohydrate in a ratio of 100: 0.75: 125: 0.75 parts by mass) undergoes a fermentation process (7 d) followed by a maturation period of six months at 15°C.

Hydroethanolic liquid extract (Weleda AG, Arlesheim, Switzerland) was produced according to the manufacturing instructions documented in Ph.Eur. method 1.1.4 (GHP 2b). In brief, comminuted fresh flowering whole plants were mixed with ethanol 62% (*m/m*) and incubated for maceration in closed containers at room temperature for 10–30 days with repeated stirring. The maceration mixture was pressed and filtered to obtain the final tincture with an ethanol concentration of 30% (*m/m*).

Arnica mother tincture (DHU-Arzneimittel GmbH & Co. KG, Karlsruhe, Germany) was manufactured according to the production method 1.1.8 documented in Ph.Eur. (GHP 4a). Therefore, in total 1 part of freshly powdered (sieve number 710) dried underground parts of *Arnica montana* L. was extracted with 10 parts of ethanol 86% (*m/m*). In a first step, the powder was thoroughly mixed with a portion of ethanol 86% (*m/m*), so that the drug material was completely soaked. The mixture was allowed to stand for approx. 24 h (used to pre-swell the drug). Then it was transferred to a percolator and allowed to percolate slowly with the remaining ethanol, at room temperature. After finalization of percolation, the residue was pressed out and the expressed liquid combined with the percolate. The combined liquids were filtrated finally. All three Arnica extracts were used in the experiments in a non-toxic 1:1,000 dilution. As Arnica extracts are a complex mixture of substances, the concentration of individual substances is not specified.

Pure 3-*O*-caffeoylquinic acid (Extrasynthese), 3,5-di-*O*-caffeoylquinic acid (Extrasynthese), 3,4,5-tri-*O*-caffeoylquinic acid (MedChemExpress), thymol (Sigma-Aldrich), helenalin (Focus Biomolecules), and TPCA1 (Abcam) were dissolved in DMSO and aliquots were stored at –80°C.

### 2.2 Primary cells and cell lines

Human PBTs were freshly isolated from heparinized whole blood of healthy donors. PBMCs were obtained by density-gradient centrifugation using FicoLite-H (Linaris). Pure PBTs were isolated from PBMCs by negative selection using Pan T cell Isolation Kit, human (Miltenyi Biotec).

PBTs and Jurkat E6.1 cells (ATCC®) were cultivated in RPMI 1640 medium (Gibco) supplemented with FCS (10% FCS, v/v, PAN-Biotec) and L-glutamine (2 mM, Gibco). Jurkat cells were subcultured thrice a week.

### 2.3 Treatment with Arnica extracts or pure compounds, *in vitro* stimulation and cell viability analysis

Before stimulation, PBTs or Jurkat cells were preincubated with test drug (Ferm, Tota, Radix, helenalin (0.25 μM) and thymol (500 μM), respectively) or vehicle (H_2_O, 0.1% EtOH or 0.1% DMSO) for 1 h at 37°C. Drug-treated samples were prepared to contain ≤ 0.1% EtOH or 0.1% DMSO for direct comparability to the solvent control.

Cells were stimulated using plate-bound anti–CD3 (20 ng/ml, clone OKT3, produced in the Section Molecular Immunology, Institute of Immunology, Heidelberg University Hospital) and anti-CD28 (75 ng/ml, clone CD28.2, BD Biosciences) antibodies. Depending on the readout, cells were stimulated for different time periods, as indicated. Cell viability was assessed by 7-AAD (BioLegend) exclusion. A threshold of ≥ 90% live cells was set to define non-toxic drug concentrations.

### 2.4 T cell proliferation assay

To track cell division, PBTs were labeled with 1 μM carboxy fluorescein succinimidylester (CFSE; Thermo Fisher Scientific) for 15 min at 37°C and stimulated for 72 h. T cell proliferation was analyzed by CFSE dilution using flow cytometry (BD™ LSRII).

### 2.5 IL-2 rescue experiment

PBTs were labeled with CFSE, left unstimulated or pretreated with Arnica extract or vehicle for 1 h and subsequently activated with anti-CD3/CD28 antibodies for 72 h in the presence or absence of 40 U/ml recombinant human IL-2. T cell proliferation was assessed by CFSE dilution.

### 2.6 T cell migration assay

The migratory capacity of T cells was assessed using Transwell™ 96-Well inserts (Corning) with 5 μm pore size. Cells placed in the upper compartment were allowed to migrate towards SDF-1α (100 ng/ml, R&D Systems) in the lower compartment for 90 min. Cells that migrated to the lower chamber were quantified by flow cytometry (BD™ LSRII). The percentage of migrated cells was calculated based on a control sample with 100% migrated cells, for which the cell suspension was added to the lower compartment.

### 2.7 Cytokine secretion assay

Twelve different cytokines (IL-2, IL-4, IL-5, IL-6, IL-9, IL-10, IL-13, IL-17A, IL-17F, IL-22, IFN–γ, and TNF–α) released by PBTs during 24 h stimulation were simultaneously quantified in cell culture supernatant using LEGENDplex™ Human Th Cytokine panel (12-plex, BioLegend) according to the manufacturer’s instructions.

### 2.8 Flow cytometry

PBT surface marker expression was analyzed after 24 h stimulation using the following directly-labeled antibodies: anti-CD3–FITC (clone SK7, BioLegend), anti-CD25–APC (clone M-A251, BioLegend), and anti-CD69–FITC (clone FN50, BioLegend). Flow cytometry was performed according to the guidelines (16).

Intracellular IL-2 was stained after 24 h of stimulation, including 4 h treatment with GolgiStop™ (BD Biosciences). PBTs were fixed with PFA and permeabilized with saponin (Sigma-Aldrich) before staining with anti-IL-2–APC antibody (clone MQ1–17H12, BioLegend).

NFκB p65 phosphorylation was assessed after stimulating PBTs for 30 min. Cells were fixed with prewarmed Cytofix™ Fixation Buffer (BD Biosciences) and permeabilized with Phosflow™Perm Buffer III (BD Biosciences). Anti-phospho-NFκB p65 (S529) (clone K10-895.12.50, BD Biosciences) antibody was used for staining.

Unbound antibodies were removed by washing three times and samples were acquired on a LSRII flow cytometer (BD Biosciences). Data analysis was performed using FlowJo software version 10 (FlowJo).

### 2.9 RNA quality control and quantification

PBTs were stimulated for 4 h and total RNA was isolated using the Direct-zol™ RNA Microprep Kit (Zymo Research) according to the manufacturer’s instructions. The quality and quantity of RNA isolated for nCounter^®^ gene expression analysis was analyzed using a NanoDrop2000c Spectrophotometer (Thermo Scientific). In addition, the RNA samples were quantified using the Qubit Fluorometer (Thermo Fisher Scientific) and RNA integrity was assessed on the Agilent 2100 Bioanalyzer (Agilent Technologies). RNA samples were stored at -80°C until further processing.

### 2.10 nCounter^®^ gene expression analysis

nCounter® target gene expression analyses were carried out at the nCounter® Core Facility on a SPRINT system by nCounter® technology (nanoString Technologies). The nCounter® technology allows for multiplexed gene expression analysis based on simultaneous hybridization and digital quantification of fluorescently-labelled oligonucleotide probes (17). The expression of 579 immune–related genes plus 15 housekeeping genes as internal reference was analyzed using the nCounter® Human Immunology V2 Panel (nanoString Technologies).

Data evaluation, background correction and normalization were carried out using the nSolver™ Analysis Software (version 4.0) provided by nanoString Technologies (https://nanostring.com/products/analysis-solutions/ncounter-analysis-solutions). NormFinder algorithm (v0.953, download of MS Excel Add-In via https://www.moma.dk/software/normfinder) was used to identify the most stable housekeeping genes among the 15 included internal reference genes based on the raw data. Genes showing a stability value below 0.5 were used for data normalization in nSolver™. Using GraphPad Prism 9, a multiple *t* test analysis (Benjamini and Hochberg) was performed to identify the significantly up- or downregulated genes with a *p*-value ≤ 0.05.

Normalized gene expression data were used for the KEGG (Kyoto Encyclopedia of Genes and Genomes, https://www.genome.jp/kegg/) pathway GSEA performed by the NGS Core Facility. GSEA was used to evaluate if defined lists (or sets) of genes exhibit a statistically significant bias in their distribution within a ranked gene list using the software packages in R, version 4.2.2. The pathway analysis was performed with fgsea package (18) and the enrichment browser package (19).

### 2.11 Analysis of nuclear translocation

Nuclear translocation of NFκB was analyzed in PBTs after 30 min of stimulation using the Amnis® NFκB Translocation Kit (Luminex Corporation) according to the manufacturer’s protocol. Fluorescent signals were detected on the Amnis® ImageStream®X Mk II (Luminex Corporation) imaging flow cytometer and data were analyzed using the IDEAS® Analysis Software (Luminex Corporation). The IκB kinase inhibitor TPCA1 (10 μM) was used as positive control. The applied gating strategy is depicted in **Supplementary Figure S7B**.

### 2.12 DNA binding assay

PBTs were stimulated for 30 min and nuclear lysates were prepared. After 15 min swelling in hypotonic buffer solution, cell membranes were disrupted by addition of Nonidet P-40 Assay Reagent (Cayman Chemical). Cytoplasmic extract was discarded and isolated nuclei were lysed in nuclear lysis buffer by rocking on a shaking platform for 30 min.

The DNA binding activity of nuclear NFκB was studied using the ELISA-based NFκB (p65) Transcription Factor Assay Kit (Cayman Chemical) according to the manufacturer’s instructions. Mean absorbance values obtained after blank subtraction were normalized to the protein concentration of nuclear lysates which was determined using the Pierce™ Coomassie Plus (Bradford) Assay Kit (Thermo Scientific) according to the manufacturer’s protocol.

### 2.13 NFAT luciferase reporter assay

Jurkat 6.1 cells were transfected with NFAT luciferase reporter plasmid based on the pGL2–Promoter Vector (NFAT-pGL2-Promoter Vector) using Human T cell Nucleofector™Kit (Lonza). The next day, cells were preincubated with drug or vehicle for 1 h and stimulated for 6 h. After the cells were lysed in lysis buffer for 15 min at 37°C, 20 μl of the lysate were incubated with 100 μl D-Luciferine-containing Beetle-Juice (PJK) for 5 min at room temperature. Luminescence was measured using a Tecan Ultra Microplate Reader. Following blank subtraction, luminescence values were normalized to the protein concentration in the lysates, which was determined using the Pierce™ 660 nm Protein Assay Kit (Thermo Scientific) according to the manufacturer’s instructions.

### 2.14 Measurement of intracellular calcium levels

Indo-1 AM dye (Thermo Fisher Scientific) was used as calcium sensor to quantify intracellular calcium levels by flow cytometry (BD LSR Fortessa™). PBTs were labeled with 2 μg/ml Indo–1 for 45 min at 37°C. Baseline calcium levels were measured for 1 min followed by addition of 20 ng/ml anti-CD3 antibody (clone OKT3, produced in Section Molecular Immunology, Institute of Immunology, Heidelberg University Hospital). The measurement was continued for 4 min, whereafter T cell activation was induced by addition of 8 μg/ml goat anti–mouse antibody (Jackson ImmunoResearch) for CD3 crosslinking. Stimulation-induced calcium influx was continuously acquired for 14 min, before maximum calcium influx was induced by addition of 4 μg/ml ionomycin (Sigma-Aldrich). After another 2 min, the analysis was stopped. Data were analyzed in FlowJo by calculating the ratio of Indo-1 AM calcium bound (~400 nm) to Indo-1 AM no calcium bound (~475 nm) signal. The results are presented as Area Under the Curve (AUC).

### 2.15 Immunoblot

Total cell lysates were prepared from PBTs after 30 min stimulation. Proteins were separated by size in a 14% SDS polyacrylamide gel followed by blotting on an Immobilon-FL PVDF membrane with 0.45 μm pore size (Merck Millipore). Total and phosphorylated proteins were detected using the following primary antibodies: anti-Cofilin (1:10,000, polyclonal, produced in Section Molecular Immunology, Institute of Immunology, Heidelberg University Hospital), anti-phospho-Cofilin (S3) (1:1,000, polyclonal, Cell Signaling), anti-L-plastin (1:1,000, clone LPL4A.1, Thermo Fisher Scientific), anti–phospho-L-plastin (S5) (1:1,000, monoclonal, produced in Section Molecular Immunology, Institute of Immunology, Heidelberg University Hospital), and anti-GAPDH (1:5,000, polyclonal, Thermo Fisher Scientific). Anti-rabbit IgG–IRDye® 800CW (1:10,000, LI-COR) and anti-mouse IgG–IRDye® 680RD (1:10,000, LI-COR) were used as secondary antibodies. The fluorescent signal was detected using the Odyssey® Imaging System (LI–COR). Signal intensity was evaluated using the Image Studio™ Lite Software (LI-COR) and normalized to the housekeeping protein GAPDH.

### 2.16 Phytochemical characterization of plant extracts

**Figure 1:**
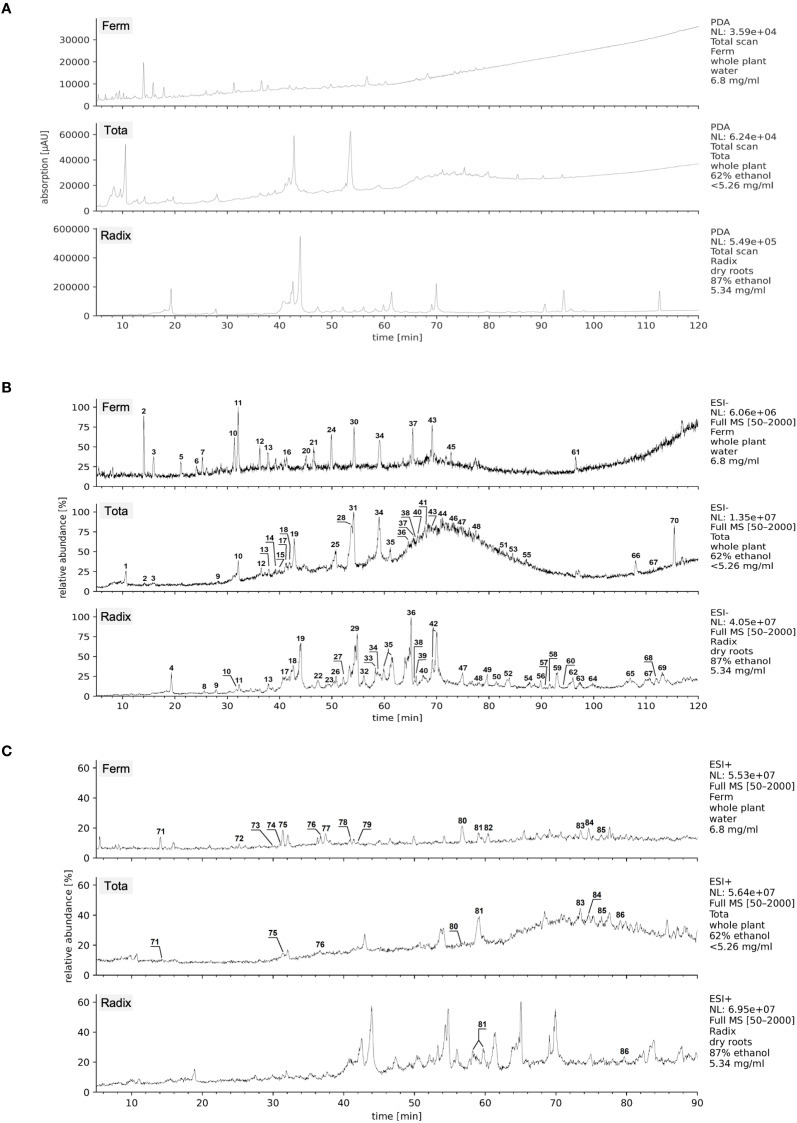
LC-MS/MS chromatograms of Arnica extracts with peak annotations. **(A)** Photodiode array (PDA) chromatograms (λ = 200–600 nm) of Ferm, Tota, and Radix extract. **(B)** Total ion currents (TICs) in ESI- mode (*m/z* = 50–2000) of Ferm, Tota, and Radix extract. For peak annotations s. **Table 1**. **(C)** TICs in ESI+ mode (*m/z* = 50–2000) of Ferm, Tota, and Radix extract. For peak annotations s. **Table 2**.

Two batches of each of the three described extracts were processed. All samples were ultrasonicated for 2 min, diluted to an approx. concentration of 5 mg dry residue/ml (end concentrations of the used extracts had a rage from 5.26 to 6.80 mg dry residue/ml compare subscript **Figure 1B**) in the corresponding solvent mixture each, and centrifuged for 10 min at 13,000 rpm before transfer to GC-vials. For dilutions the solvent compositions according to the manufacturer’s instructions were used each. One exception was made in the case of the extract Tota. Here, the higher ethanolic content of 62% of the initial extraction conditions were used (compare above) to ensure the maximum solubility of the containing compounds.

LC-MS/MS analysis was performed on a Finnigan LCQ-Duo ion trap mass spectrometer with an Electrospray Ionization (ESI) source (ThermoQuest) coupled to a Thermo Scientific Accela HPLC system (MS pump plus, autosampler, and PDA detector plus) (ThermoQuest) with an EC 150/3 Nucleodur 100–3 C18ec column (Macherey-Nagel). A gradient of water and acetonitrile (ACN) with 0.1% formic acid each was applied from 5% to 30% ACN in 60 min and to 95% ACN in another 60 min at 30°C. The flow rate was 0.5 ml/min and the injection volume was 20 µl. All samples were measured in ESI+ and ESI- mode. The MS was operated with a capillary voltage of 10 V (ESI+) or -10 V (ESI-), source temperature of 240°C, and high purity nitrogen as a sheath and auxiliary gas at a flow rate of 80 and 40 (arbitrary units), respectively. The ions were detected in a mass range of m/z 50–2000. The collision energy of 35% was used in MS2 for fragmentation. The PDA absorption data were measured as sum spectra between 200–600 nm. Data acquisitions and analyses were carried out by Xcalibur™ 2.0.7 software (Thermo Scientific).

The batches of each extract showed an identical composition; therefore, only one batch of each extract was used for all further investigations. The resulting data of the chosen batch samples from the three different Arnica extracts (**Figure 1**) were edited manually. The phytochemical compounds were determined by tentative identification based on the given literature and own calculations on base of the fragmentation patterns. In this context, the ESI- results were used mostly for the determination of the caffeoylquinic acid derivatives (**Table 1**, **Supplementary Table S1**) and the ESI+ results for the sesquiterpene lactones (helenalins and dihydrohelenalins, **Table 2**).

**Table 1:** LC-MS/MS results (ESI-) of Arnica extracts with tentative identification of phytochemical compounds (20–27).

Peak No.	Retention time, t_R_[min]^*^	*m/z*		Tentative identification	Occurrence in Arnica extracts**		
		Precursor ion (ESI-)	Main product ion MS2		Ferm	Tota	Radix
1	7.9–10.8	353.0	191.2	Caffeoylquinic acid derivative (20, 21)		x	
2	14.0–14.3	443.1	381.2	N/A	x	x	
		489.0	443.1		**x**	**x**	
3	15.0–16.2	197.1	161.0	Trihydroxythymol (22)	x	x	(x)
4	18.2–19.9	353.1	191.2	Caffeoylquinic acid derivative (20, 21)			x
		707.0	352.8	Caffeoylquinic acid derivative dimer			**x**
5	21.0–21.3	489.1	425.1	N/A	x		
6	24.1–24.4	165.1	147.0	N/A	x		
7	25.1–25.4	473.2	427.1	N/A	x		
8	25.1–25.8	463.3	331.1	N/A			x
9	27.2–28.2	515.2	353.1	Dicaffeoylquinic acid derivative (21)		x	x
10	29.9–32.3	471.1	425.1	N/A	x	x	
		525.1	481.3			**x**	x
		611.3	431.1			x	
11	31.9–32.4	525.1	481.3	N/A	x		x
12	35.8–36.6	449.2	269.1	N/A	x	x	
13	37.4–38.7	373.1	329.1	N/A	x	x	x
		535.1	373.1				**x**
14	38.8–39.4	477.1	301.1	Quercetin-glucuronide/ Quercitin-*O*-rhamnoside isomer (23, 24)		x	
15	39.7–40.3	515.2	352.9	Dicaffeoylquinic acid derivative (21)		x	
		605.3	561.3	N/A		**x**	
16	41.2–41.5	287.2	269.1	N/A	x		
17	40.2–41.6	515.1	353.0	Dicaffeoylquinic acid derivative (21)		x	x
18	41.6–43.0	515.1	353.0	Dicaffeoylquinic acid derivative (21)		x	x
19	42.2–44.4	515.1	353.0	Dicaffeoylquinic acid derivative (21)		x	x
20	44.8–45.4	231.3	213.1	N/A	x		
21	46.3–46.9	217.3	171.2	N/A	x		
22	47.0–47.7	515.1	353.1	Dicaffeoylquinic acid derivative (21)			x
23	48.7–49.8	677.2	497.2	Tricaffeoylquinic acid derivative (20, 25, 26)			x
24	49.7–50.2	447.3	315.1	N/A	x		
		493.1	447.2		**x**		
25	50.1–51.0	471.1	425.0	N/A		x	
		517.1	471.0			**x**	
26	50.2–51.0	493.2	447.2	N/A			**x**
		677.2	497.2	Tricaffeoylquinic acid derivative (20, 25, 26)			x
27	51.6–52.5	677.2	497.2	Tricaffeoylquinic acid derivative (20, 25, 26)			x
28	52.8–53.9	207.2	179.3	Ethyl caffeate (22, 27)		x	
29	53.0–55.0	609.2	522.9	N/A			x
30	53.7–54.5	609.1	565.5	N/A	x		
31	53.9–54.6	609.2	491.2	N/A		x	
32	55.4–57.1	583.2	421.0	N/A			x
33	57.1–59.1	583.2	421.0	N/A			x
34	56.8–59.8	266.9	161.1	N/A	x	x	
		689.3	645.4		**x**	**x**	x
35	59.1–62.1	609.2	565.2	N/A		**x**	
		609.1	485.9				x
		677.2	515.1	Tricaffeoylquinic acid derivative (20, 25, 26)		x	**x**
36	63.5–65.3	565.4	463.3	N/A		x	x
		611.1	459.9	N/A		**x**	
37	65.3–65.7	327.3	291.3	N/A	x	x	
38	65.6–66.0	609.2	565.3	N/A		x	
		609.2	489.2	Lucenin-2 (25)			x
39	66.0–66.31	609.2	447.11	N/A			**x**
		677.1	515.1	Tricaffeoylquinic acid derivative (20, 25, 26)			x
40	66.1–66.6	299.2	284.2	Hispidulin (23)		x	
41	67.2–68.5	281.0	161.0	N/A		x	
		645.5	543.4	N/A		x	x
		785.6	665.3	N/A		x	
42	68.7–74.1	745.2	583.0	N/A			x
43	68.7–69.7	329.3	229.3	N/A	x	x	
44	71.0–71.3	785.5	665.3	N/A		x	
45	72.6–72.9	329.3	311.2	N/A	x		
46	73.0–73.2	477.2	369.2	N/A		x	
47	74.6–75.0	693.2	531.1	N/A		x	
			649.3	N/A			x
48	77.0–78.6	713.4	681.3	N/A		x	
			698.2				x
49	79.0–79.8	649.5	547.3	N/A			**x**
		695.0	528.6	N/A			x
50	81.0–81.9	649.4	547.3	N/A			x
51	82.5–82.8	491.2	313.1	N/A		x	
52	83.2–84.0	337.0	161.1	N/A			x
53	84.4–84.7	601.5	431.2	N/A		x	
54	87.0–88.0	351.0	161.1	N/A			x
55	88.6–89.1	489.3	474.1	N/A		x	
56	89.6–90.1	476.4	279.3	N/A			x
57	90.3–91.2	649.2	603.0	N/A			x
58	91.3–91.9	564.3	504.2	N/A			x
59	92.5–93.5	564.3	504.3	N/A			x
60	93.7–95.0	595.4	415.2	N/A			x
61	96.5–96.8	416.1	nl	N/A	x		
62	95.0–96.6	595.4	415.2	N/A			x
63	96.8–98.1	540.3	480.3	N/A			x
64	98.5–102.5	571.4	391.2	N/A			x
65	105.8–108.2	433.3	153.1	N/A			x
66	107.8–108.6	297.3	297.3	N/A		x	
67	109.6–111.2	529.3	441.1	N/A			x
		730.6	326.2	N/A			x
		776.3	729.5	N/A			**x**
68	111.8–112.2	379.4	361.2	N/A			x
69	112.5–113.9	776.3	729.0	N/A			x
70	115.2–115.7	339.3	297.3	N/A		x	

Ferm, aqu. total ferm; Tota, ethanolic total extract; Radix, ethanolic root tincture. *t_R_ is based on the MS data. Delay time 0.06–0.09 min; t_0_ was not subtracted (1.85 min in PDA). **bold x marks the dominant compound ion of the corresponding peak; (x): shifted and spread over a wider area; N/A, not available; nl, neutral loss. More detailed information in **Supplementary Table S1**.

**Table 2:** LC-MS/MS results (ESI+) of Arnica extracts with tentative identification referring to sesquiterpene lactones as well as thymol derivatives (5, 7, 22, 28–30).

Peak no.	Retention time, t_R_[min]*	*m/z*		Tentative identification	Occurrence in Arnica extracts		
		Precursor ion (ESI+)**	Product ions MS2**		Ferm	Tota	Radix
71	14.01–14.16	265.0	148.2 (21), 161.2 (26), **190.5** (50), 201.2 (38), 205.1 (23), **219.0** (100), 220.6 (27), **222.6 (92)**, **246.9 (98)**, **251.53 (58)**, 252.86 (34)	Dihydrohelenalin isomer (28)	x	x***	
		**282.9**	**247.0 (58)**, **264.9 (100)**	Dihydrohelenalin + H_2_O (29)			
72	25.05–25.35	249.0	157.1 (14), **175.1 (50)**, 185.1 (14), **203.0 (47)**, **231.0 (100)**	Dihydroaromaticin isomer (5)	x		
73	29.88–30.03	249.0	195.0 (18), 213.0 (37), **230.9 (100)**	Dihydroaromaticin isomer (5)	x		
74	30.89–31.14	249.0	194.9 (3), 213.1 (16), **230.9 (100)**	Dihydroaromaticin isomer (5)	x		
75	31.19–31.54	264.9	201.0 (20), 219 (22), 229.0 (25), **247.0 (100)**	Dihydrohelenalin isomer (28)	x	x***	
76	36.56–36.86	247.0	173.1 (35), **201.0 (82)**, **229.0 (100)**	Dihydrohelenalin isomer - H_2_O	x	x***	
		**264.9**	201.0 (17), 219.0 (17), 229.0 (21), **247.0 (100)**	Dihydrohelenalin isomer (28)			
77	37.21–37.76	**265.0**	201.0 (10), 219.0 (27), 229.0 (27), **247.0 (100)**	Dihydrohelenalin isomer (28)	x		
		324.9	203.0 (27), 221.1 (34), **247.0 (76)**, **264.9 (100)**, 282.3 (21), 323.4 (13)	Chamissonolide (5)			
78	40.67–41.13	262.9	199.1 (12), 219.0 (13), 227.0 (30), **245.0 (100)**	Helenalin isomer (5, 28)	x		
79	42.02–42.17	263.0	189.0 (9),199.1 (18), 201.1 (9), 217.0 (22), 227.0 (45), 235.0 (18), **245.0 (100)**	Helenalin isomer (5, 28)	x		
80	56.50–57.04	247.1	159.1 (14), 173.0 (43), 183.1 (16), **201.0 (67)**, 219.0 (12), **229.0 (100)**	Dihydrohelenalin acetate - acetic acid [M+H-C_2_H_4_O_2_]^+^ (29)	x	x***	
		306.9	**247.0 (100)**, 287.6 (18)	Dihydrohelenalin acetate (7, 29, 30)			
81	58.46–59.64	251.0	145.1 (100), 162.9 (99)	10-Hydroxy-8,9-epoxy-thymolisobutyrate (22)	x	x	(x)
82	60.15–60.55	304.9	227.1 (14), **245.0 (100)**, 262.9 (19)	Helenalin acetate (28, 29)	x		
83	73.18–73.74	332.9	229.0 (7), **247.0 (100)**, 287.5 (30)	Dihydroxyhelenalin methacrylate (28)	x***	x	
84	74.44–74.75	334.9	229.2 (6), **247.0 (100)**, 265.2 (5), 287.2 (25)	Dihydrohelenalin isobutyrate (5)	x	x	
85	76.26–76.66	333.0	209.3 (4), 227.0 (15), **245.0 (100)**, 263.0 (23), 285.4 (10)	Helenalin isobutyrate (28)	x***	x***	
86	78.95–79.11	316.2	245.26 (3), 262.79 (5), **280.23 (48)**, **298.12 (100)**, 299.12 (3)	4-Hydroxy-8-sphingenine (22)		x	(x)***

Ferm, aqu. total ferm; Tota, ethanolic total extract; Radix, ethanolic root tincture. *t_R_ is based on the MS data. Delay time 0.06–0.09 min; t_0_ was not subtracted (1.85 min in PDA); **bold molecule ion masses mark the dominant compound ion of the corresponding peak or the main fragment ions of the corresponding molecule ion, respectively. ***smaller amounts; (x) shifted and/or spread over a wider area.

The thymol contents of the Arnica extracts were investigated by thin-layer chromatography (TLC) after Rossetti et al. (31) with a few adjustments. Briefly, a silica gel 60 F_254_ TLC plate (Merck) and cyclohexane:diethyl ether (70:30) as mobile phase were used for separation. As reference, 5 µg thymol were applied; 20 µl of the extracts were used. The test substances were applied as bands of 15 mm and the plate was developed over a path of approx. 7.5 cm. The plate was treated with anisaldehyde solution R, heated at 100°C for 10 min, and examined at daylight.

### 2.17 Statistics

Statistical analyses were performed using the GraphPad Prism 9 software. The data are presented as mean ± SEM. Significant numerical differences compared to the respective solvent control sample were determined based on a one-way Analysis of Variance (ANOVA) or a Mixed-effects analysis in case of missing values with Dunnett´s *post-hoc* test. Differences with a *p*-value ≤ 0.05 were considered as being statistically significant (*p ≤ 0.05; **p ≤ 0.01; ***p ≤ 0.001; ****p ≤ 0.0001). Only experiments showing a significant difference between the unstimulated sample and the stimulated H_2_O control were evaluated. Therefore, significances for this comparison are not indicated in the graphs.

## 3 Results

### 3.1 Differentially prepared Arnica extracts differ in their phytochemical composition

Plant extracts represent a complex mixture of different bioactive ingredients. In order to gain insight into which substances are most abundant and may be primarily responsible for the effects induced by the extract, the three Arnica extracts were characterized by LC-MS/MS with subsequent tentative identification of phytochemical compounds. The photodiode array (PDA) chromatograms of the extracts (**Figure 1A**) already indicate major differences in the phytochemical composition. These differences become even more obvious in the total ion chromatograms (TICs) of the negative mode summarized in **Figure 1B**. These differences in composition can be attributed to different solvents, extraction methods, and also to different plant parts. While the aqueous Ferm extract (top) contains mainly hydrophilic compounds, the hydroethanolic Tota extract (middle) also includes hydrophobic substances that elute later in the HPLC chromatogram. Ferm and Tota were both prepared from plant material of the whole plant, whereas for Radix (bottom) only the underground parts and solvent with higher ethanol content were used, resulting in a different composition with more hydrophobic compounds compared to the other two extracts.

**Figure 2:**
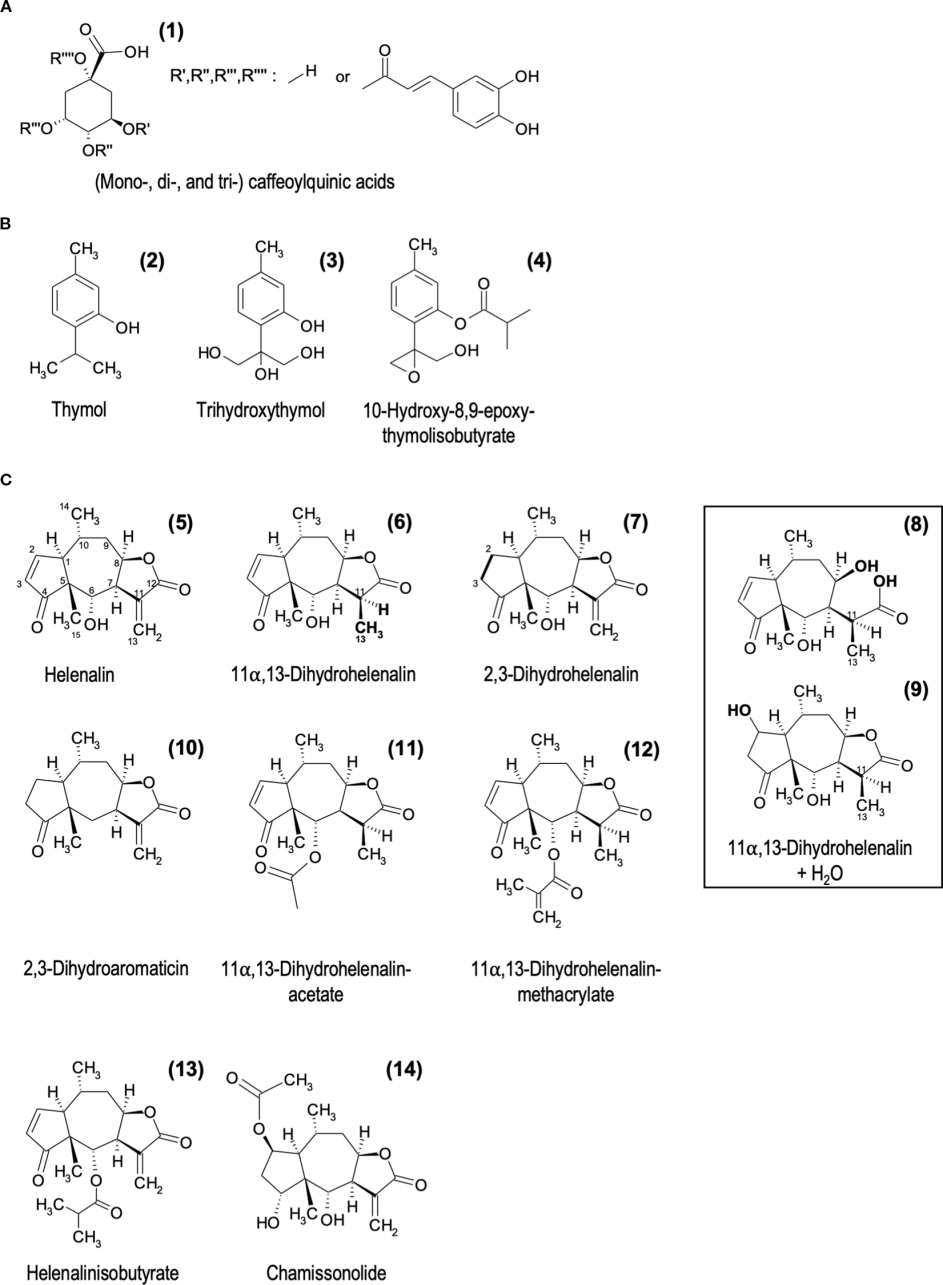
Chemical structures of the most important phytochemical compound groups determined in the investigated Arnica extracts by LC-MS/MS and TLC. **(A)** Caffeoylquinic acid derivatives [(1) ESI-, **Figure 7** + **Table 1** + **Supplementary Table S1**], **(B)** Monoterpenes [(2) TLC, **Supplementary Figure S8** (3); ESI-, **Figure 7** + **Table 1**+ **Supplementary Table S1** (4); ESI+, **Figure 8** + **Table 2**), **(C)** Sesquiterpene lactones ( (5–12) ESI+, **Figure 8** + **Table 2**].

Probably due to the treatment of the plant material during the extraction procedures, it was not possible to determine a large number of the detected compounds by tentative identification based on the available literature (**Table 1**, **Supplmentary Table S1**). Nevertheless, (mono-, di-, and tri-) caffeoylquinic acid derivatives [**Figure 2A** (1)] could be identified as one of the main substance groups in Tota and Radix. The highest levels of these compounds were found in Radix.

For the determination of sesquiterpene lactones, the positive mode was used. A selected overview is given in **Figure 1C** and **Table 2**. The chemical structures of the determined compounds are shown in **Figure 2C** (5–14). The highest levels of helenalins and dihydrohelenalins were found in the Ferm extract. Lower concentrations and a smaller number of sesquiterpene lactone derivatives were found in Tota, with the exception of dihydrohelenalin methacrylate [**Figure 2C** (12)]. In Radix, almost no sesquiterpene lactone derivatives could be identified.

A different picture emerges for thymol [**Figure 2B** (2)] and the detected thymol derivatives [**Figure 2B** (3, 4)]. Trihydroxythymol [detected in ESI- (3)] and 10-hydroxy-8,9-epoxy-thymolisobutyrate [detected in ESI+ (4)] are present in all three investigated extracts. Thymol and related lipophilic terpenoids (determined by TLC, **Supplementary Figure S1**) are mainly present in Radix and in lower concentrations in Tota. In contrast, no thymol was detected in Ferm (detection limit 0.2 µg). Trihydroxythymol showed a higher estimated content in Ferm, while 10-hydroxy-8,9-epoxy-thymolisobutyrate was more prominent in Tota.

In summary, the main compounds detected in Ferm were sesquiterpene lactones, while caffeoylquinic acid derivatives and thymol were enriched in Radix. Tota showed a mixture of all substance classes, but with lower content. The mentioned thymol derivatives were found in all three extracts with different concentrations. All extracts contained additional compounds that could not be identified.

### 3.2 Arnica extracts inhibit activation and proliferation of primary human T cells

**Figure 3:**
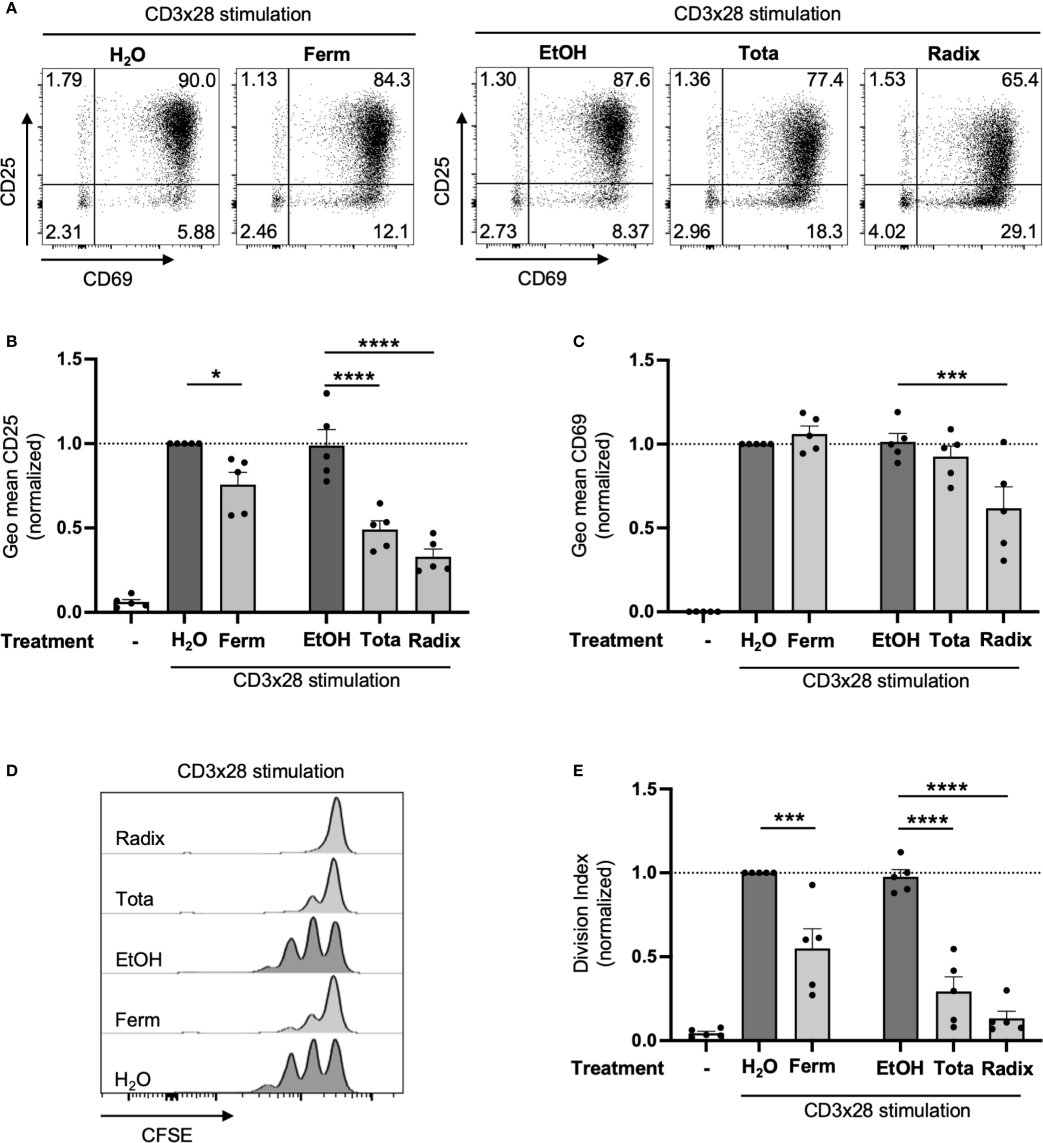
Arnica extracts inhibit activation and proliferation of primary human T cells. PBTs were left unstimulated (left white bar) or pretreated with Arnica extract (light grey bars) or vehicle (dark grey bars) for 1 h and subsequently activated with anti-CD3/CD28 antibodies. **(A–C)** After 24 h, surface expression of CD25 and CD69 was analyzed by flow cytometry. Representative dot plots including percent values **(A)** and statistical evaluation of CD25 **(B)** and CD69 **(C)** MFI from five independent experiments. **(D, E)** T cell proliferation was assessed by CFSE dilution following 72 h stimulation. Representative histograms **(D)** and statistical evaluation **(E)** from five independent experiments. Each data point represents an individual T cell donor. Data were normalized to the H_2_O control sample and are expressed as mean ± SEM. *p ≤ 0.05; ***p ≤ 0.001; ****p ≤ 0.0001.

To analyze the effects of Arnica preparations on T cell functions, three differently prepared Arnica extracts were selected and their effect on PBTs were directly compared. PBTs were preincubated with a non-toxic 1:1,000 dilution (**Supplementary Figure S2A**) of each extract or vehicle and then co-stimulated with anti-CD3 and anti-CD28 antibodies (CD3xCD28 stimulation). This mode of activation mimics antigen recognition on APCs via the TCR and the costimulatory receptor CD28. T cell activation is reflected by upregulation of the surface expression of CD25 and CD69. Compared to the H_2_O vehicle control, aqueous fermented whole plant extract (Ferm) significantly diminished the mean fluorescent intensity (MFI) of CD25 per cell. An even stronger inhibition was observed upon treatment with hydroethanolic whole plant extract (Tota) and root mother tincture (Radix) compared to the ethanol (EtOH) vehicle control (**Figures 3A, B**). Thereby, the percentage of CD25^+^ cells was significantly diminished only by Radix extract (**Supplementary Figure S2B**). Radix extract, in addition, diminished the MFI of CD69 (**Figures 3A, C**) and the percentage of CD69–expressing PBTs (**Supplementary Figure S2C**). Such an effect was not seen upon treatment with Ferm or Tota extract.

CD25 expression is directly linked to T cell proliferation, as it represents a subunit of the receptor for the autocrine T cell growth factor interleukin-2 (IL-2Rα chain), necessary for formation of a high affinity IL-2R. Fitting to diminished CD25 surface levels, all three tested Arnica extracts inhibited T cell proliferation (**Figures 3D, E**). As observed for CD25 expression, the Tota and Radix extracts proved to be more effective in the inhibition of T cell proliferation than the Ferm extract.

### 3.3 Arnica extracts inhibit IL-2 production, but T cell proliferation cannot be rescued by IL-2 supplementation

**Figure 4:**
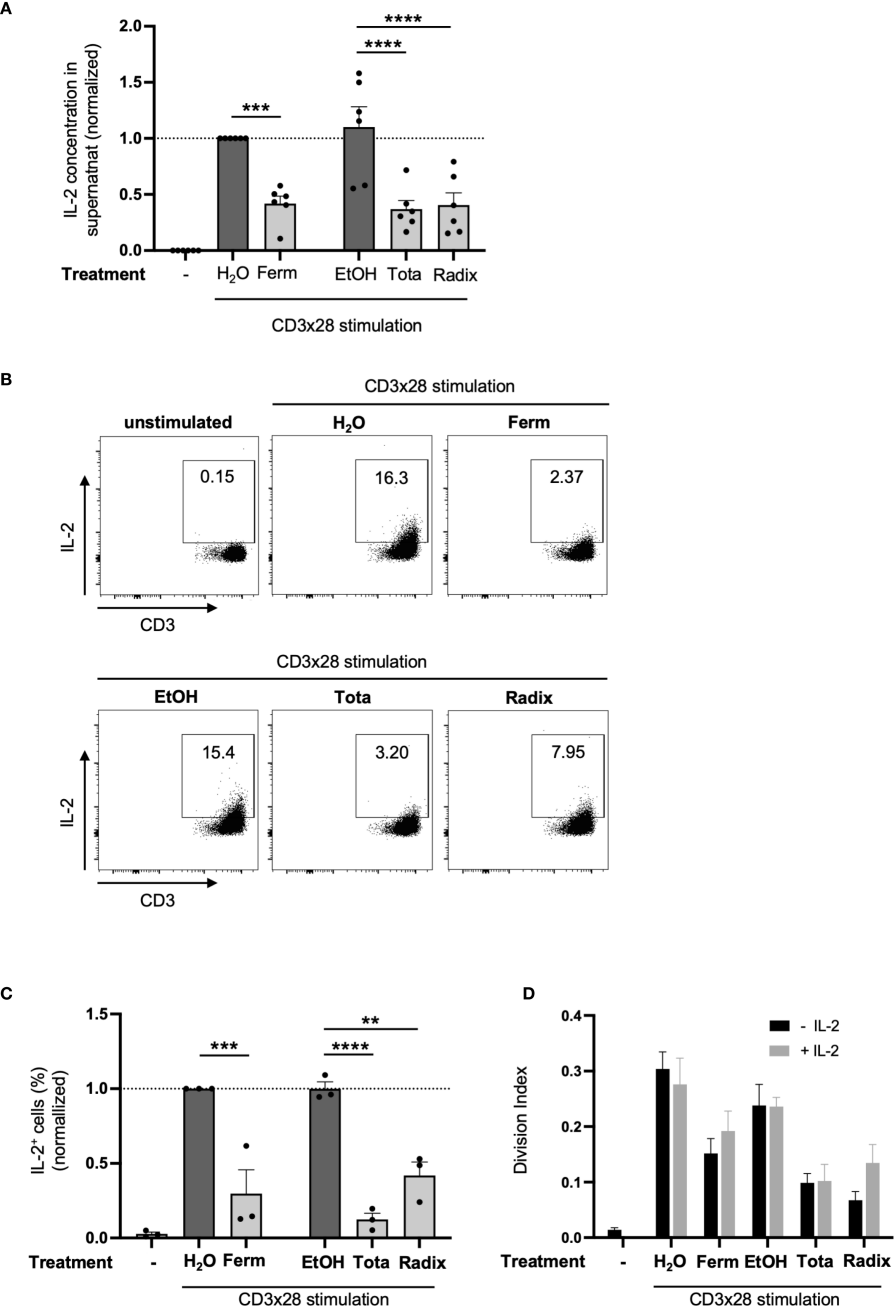
Arnica extracts inhibit IL-2 production but T cell proliferation cannot be rescued by IL-2 supplementation. **(A–C)** PBTs were left unstimulated (left white bar) or pretreated with Arnica extract (light grey bars) or vehicle (dark grey bars) for 1 h and subsequently activated with anti-CD3/CD28 antibodies for 24 (H) **(A)** IL-2 released to the supernatant was quantified by cytometric bead array (LEGENDplex™) in six independent experiments. Each data point represents an individual T cell donor. **(B, C)** Intracellular IL-2 staining analyzed by flow cytometry. Representative dot plots **(B)** and statistical evaluation of the percentage of IL-2 expressing cells **(C)** from three independent experiments. Each data point represents an individual T cell donor and data were normalized to the H_2_O control sample. **(D)** PBTs were labeled with CFSE, left unstimulated or pretreated with Arnica extract or vehicle for 1 h and subsequently activated with anti–CD3/CD28 antibodies for 72 h in the presence (grey bars) or absence (black bars) of 40 U/ml recombinant human IL-2. T cell proliferation was assessed by CFSE dilution in five independent experiments. Data are expressed as mean ± SEM. **p ≤ 0.01; ***p ≤ 0.001; ****p ≤ 0.0001.

In addition to expressing CD25, T cells need to produce the autocrine growth factor IL-2 to be able to proliferate. Therefore, IL-2 released by PBTs with or without prior drug treatment was quantified in cell culture supernatants by LEGENDplex™. As expected, CD3xCD28 stimulation resulted in an increased IL-2 content in the supernatants compared to unstimulated controls. Ferm, Tota and Radix extracts each significantly decreased the concentration of IL-2 in the supernatant to a similar extent (**Figure 4A**). In order to clarify if extract treatment inhibits cytokine production or rather its export, intracellular staining of IL-2 was conducted and analyzed via flow cytometry. As depicted in **Figures 4B, C**, the three Arnica preparations significantly diminished the percentage of PBTs containing intracellular IL-2. Thereby it was confirmed that the extracts interfere with IL-2 production.

To address the question of whether reduced IL-2 production in the presence of Arnica extracts has a decisive effect on T cell proliferation, a rescue experiment was performed. Therefore, the cell culture medium was supplemented with recombinant human IL-2. However, exogenously added IL-2 could not restore the normal cell division rate (**Figure 4D**). Note, that the functionality of the exogenously added IL-2 was confirmed by its proliferation-inducing effect on phytohaemagglutinin (PHA)-treated PBTs (**Supplementary Figure S2D**). Thus, inhibition of IL-2 responsiveness through reduced induction of the IL-2 receptor (CD25) appeared to be the primary reason for diminished T cell proliferation in the presence of the three Arnica preparations.

### 3.4 Effects of Arnica extracts on human PBTs are not mimicked by pure (mono-, di-, or tri-) caffeoylquinic acid

To determine whether caffeoylquinic acids were responsible, at least in part, for mediating the effects of Tota and Radix, they were tested in pure form for their effect on PBT function. A titration of each drug was performed to find the optimal concentration range. 3–*O*–Caffeoylquinic acid (3-CQA), also known as chlorogenic acid, had no significant effect on CD25 (**Supplementary Figure S3A**) and CD69 expression (**Supplementary Figure S4A**) after 24 h of stimulation. None of the tested drug concentrations (10–100 μM) had a cytotoxic effect at this time point (**Supplementary Figure S4B**). T cell proliferation (72 h stimulation) was also not significantly altered by 3-CQA (**Supplementary Figure S3B**). The highest non-toxic concentration after 72 h of stimulation was 25 μM (**Supplementary Figure S4B**).

Similarly, 3,5-Di-*O*-caffeoylquinic acid (3,5-diCQA) treatment (10–100 μM) was analyzed for its effect on T cell activation marker expression and proliferation. In contrast to Arnica extracts, 10 μM and 25 μM 3,5-diCQA even resulted in increased CD25 expression compared to DMSO control. The other test concentrations had no significant effect (**Supplementary Figure S3C**). 10 μM 3,5-diCQA also enhanced CD69 expression, while no significant change was observable with 25–100 μM (**Supplementary Figure S4C**). None of the tested 3,5-diCQA concentrations affected the proliferative capacity of T cells significantly (**Supplementary Figure S3D**). Compared to 3-CQA, 3,5-diCQA was found to be slightly more toxic to PBTs. After 24 h of stimulation, up to 75 μM 3,5-diCQA did not decrease cell viability below 90%, whereas after 72 h, the highest non-toxic concentration was defined as 25 μM (**Supplementary Figure S4D**).

Due to appreciably higher cytotoxicity compared to mono- and di-caffeoylquinic acid, the concentration range for testing the effect of 3,4,5-tri-*O*-caffeoylquinic acid (3,4,5-triCQA) on PBTs had to be adjusted to 0.5–5 μM. Analyzing its influence on CD25 (**Supplementary Figure S3E**) and migration (**Supplementary Figure S4E**) surface expression revealed no significant effects. Only the cytotoxic 5 μM concentration of 3,4,5-triCQA led to a significant reduction of T cell proliferation (**Supplementary Figure S3F**). Unlike 24-h assays, for which up to 2.5 μM 3,4,5-triCQA were non-toxic, a maximum of 1 μM was reliable for 72 h experiments (**Supplementary Figure S4F**). Thus, the effects of Arnica extracts on the activation and proliferation of human PBTs were not mimicked by pure (mono-, di-, or tri-) caffeoylquinic acid.

### 3.5 Thymol and helenalin inhibit CD25 expression, IL-2 production and T cell proliferation similar to the Arnica extracts

**Figure 5:**
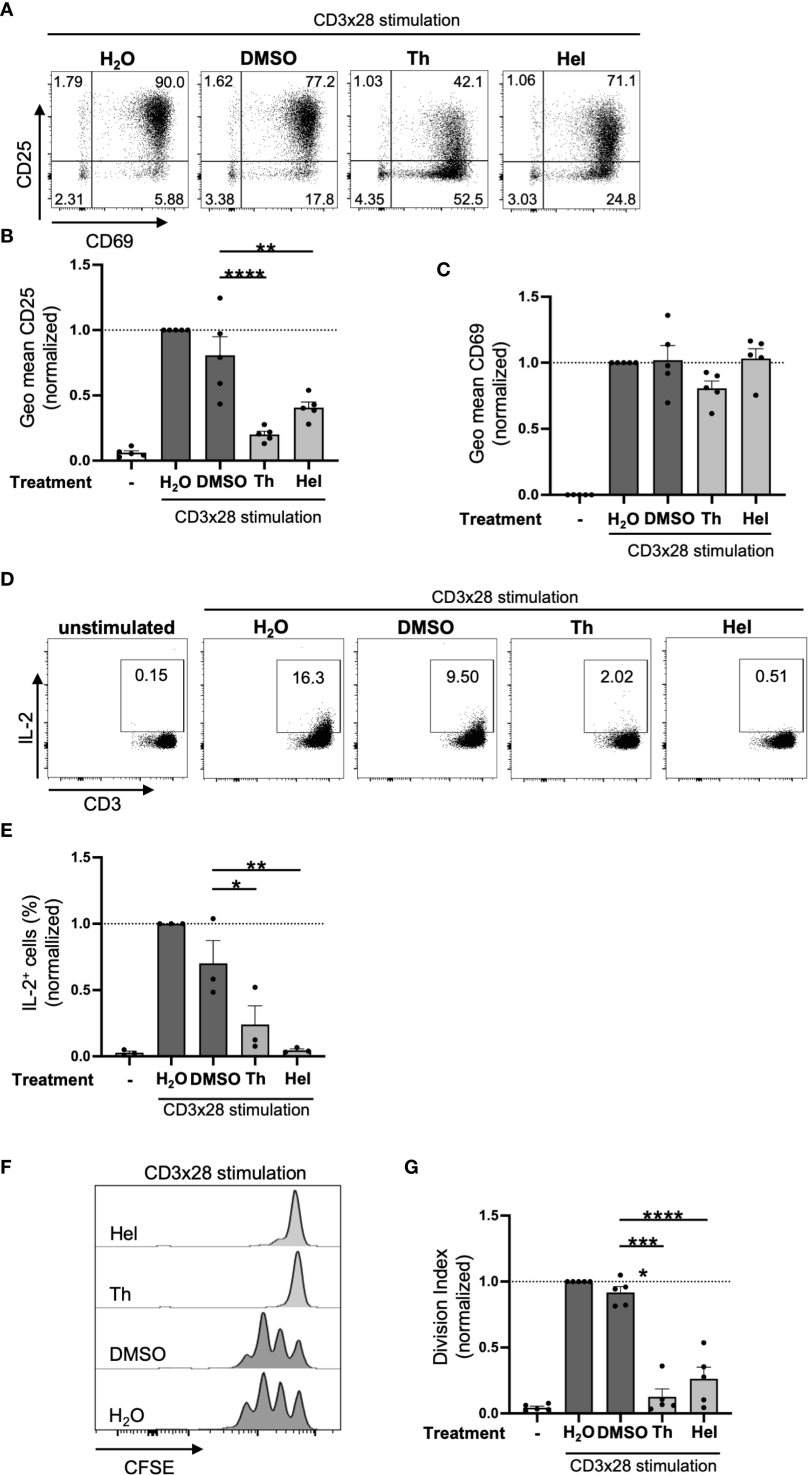
Thymol and helenalin inhibit CD25 expression, IL-2 production and T cell proliferation similar to the Arnica extracts. PBTs were left unstimulated (left white bar) or pretreated with substance (light grey bars) or vehicle (dark grey bar) for 1 h and subsequently activated with anti-CD3/CD28 antibodies for 24 (H) **(A–C)** Surface expression of CD25 and CD69 was analyzed by flow cytometry after 24 h stimulation. Representative dot plots including percent values **(A)** and statistical evaluation of CD25 **(B)** and CD69 **(C)** MFI from five independent experiments. **(D, E)** Intracellular IL-2 staining analyzed by flow cytometry after 24 h stimulation. Representative dot plots **(D)** and statistical evaluation **(E)** from three independent experiments. The data in **(D)** were obtained alongside those shown in **Figure 3B**. The same dot plots as in **Figure 3B** are therefore shown in **(D)** for unstimulated cells and for the H_2_O control of CD3xCD28-stimulated cells. **(F, G)** T cell proliferation was assessed by CFSE dilution after 72 h stimulation. Representative histograms **(F)** and statistical evaluation **(G)** from five independent experiments. Each data point represents an individual T cell donor. Data were normalized to the H_2_O control sample and are expressed as mean ± SEM. *p ≤ 0.05; **p ≤ 0.01; ***p ≤ 0.001; ****p ≤ 0.0001.

We then compared the effects of the pure compounds thymol (Th) and helenalin (Hel) on the activation of purified human PBTs with those found for the different Arnica extracts. To this end, PBTs were preincubated with helenalin (0.25 μM) or thymol (500 μM), or the respective solvent control (DMSO) and then co-stimulated with anti-CD3 and anti-CD28 antibodies (CD3xCD28 stimulation). Both, helenalin and thymol, significantly inhibited the expression of the IL-2 receptor subunit CD25 (**Figures 5A, B**). These effects are similar to the effects observed after treatment with the different Arnica extracts (compare **Figures 2**, **3**). The expression of CD69, which was significantly reduced by Radix (compare **Figure 2C**) was also reduced by thymol, albeit only by trend (**Figures 5A, C**). Similar to Ferm (compare **Figure 2C**), helenalin did not affect the expression of CD69 on PBTs.

All Arnica extracts significantly reduced the percentage of IL-2^+^ cells after 24 h of CD3x28 stimulation (compare **Figures 3B, C**). The same effect was observed after treatment with thymol or helenalin upon CD3xCD28 costimulation (**Figures 5D, E**). Consistent with this finding and similar to Arnica extracts (compare **Figures 2D, E**), treatment with thymol and helenalin resulted in decreased T cell proliferation (**Figures 5F, G**). Note, that none of these substances affected the cell viability (**Supplementary Figure S5**). Again, T cell proliferation could not be rescued by exogenous addition of IL-2 (data not shown).

### 3.6 Arnica extracts, thymol and helenalin do not affect the costimulatory signals LPL phosphorylation and cofilin dephosphorylation

**Figure 6:**
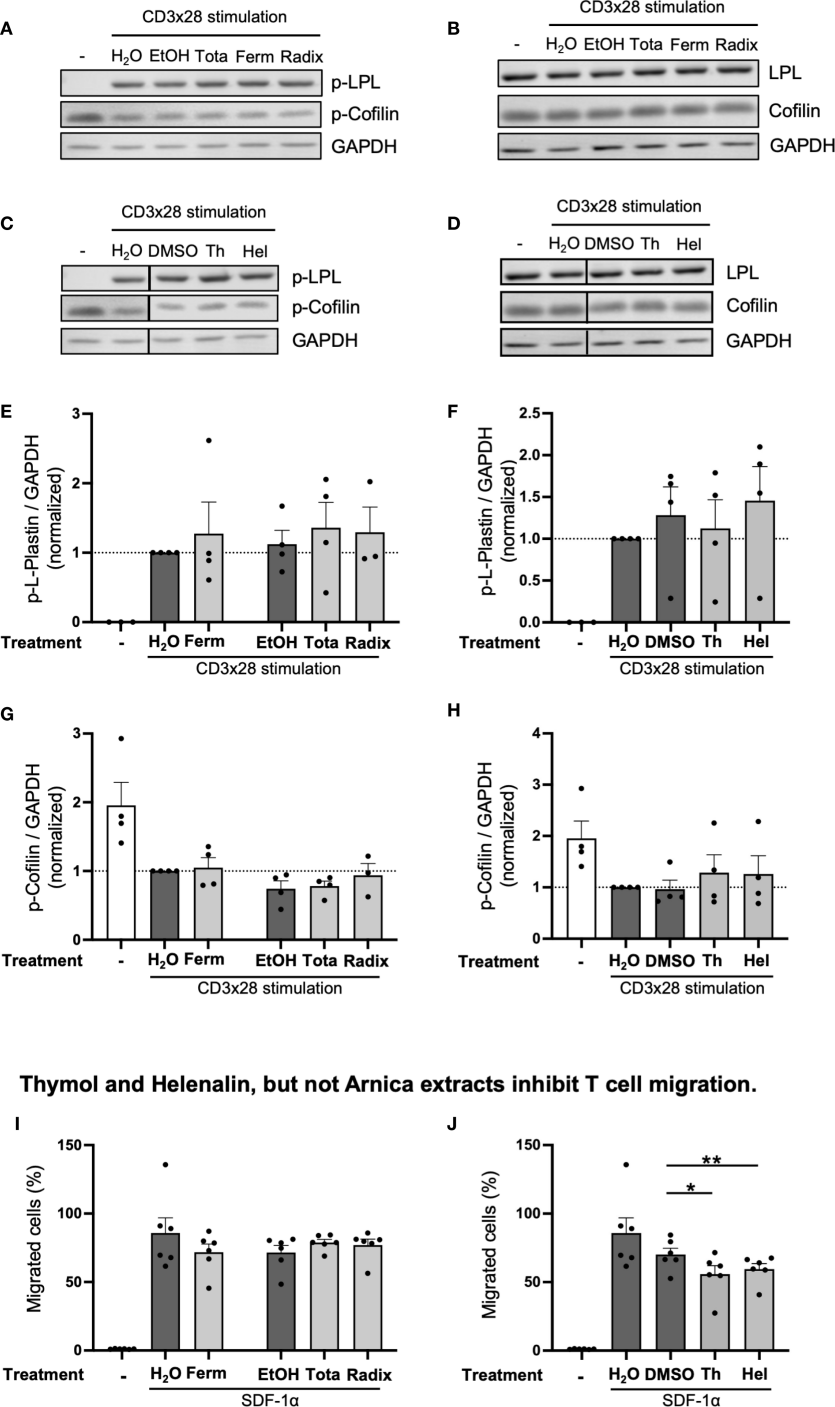
Arnica extracts, thymol and helenalin do not affect LPL phosphorylation and Cofilin dephosphorylation. Thymol and helenalin, but not Arnica extracts inhibit T cell migration. **(A–H)** PBTs were left unstimulated (left white bar) or pretreated with drug (light grey bars) or vehicle (dark grey bars) for 1 h and subsequently activated with anti-CD3/CD28 antibodies for 30 min. Phosphorylated and total LPL or cofilin were quantified by western blot. Representative blots **(A–D)**. The data in **(A)** were collected alongside with those shown in **(C)**. The data in **(B)** were collected alongside with those shown in **(D)**. Therefore, (A/C) and (B/D) respectively show the same western blots for unstimulated cells and for the H_2_O control of CD3xCD28-stimulated cells. Statistical evaluation of p-LPL **(E, F)** and p-cofilin bands **(G, H)** from four independent experiments. Each data point represents an individual T cell donor. Data were normalized to the H_2_O control sample and are expressed as mean ± SEM. **(I, J)** PBTs were left untreated (left white bar) or pretreated with drug (light grey bars) or vehicle (dark grey bars) for 1 h and transferred to the upper compartment of a 5 μm pore size Transwell™ insert. SDF-1α (100 ng/ml) was used as a chemoattractant in the lower compartment. T cell migration after treatment with Arnica extracts **(I)** or pure compounds **(J)** was quantified by flow cytometry in six independent experiments. Each data point represents an individual T cell donor. Data are expressed as mean ± SEM. *p ≤ 0.05; **p ≤ 0.01.

T cell costimulation induces intracellular costimulatory signals that are required for dynamic rearrangement of the actin cytoskeleton, formation of a mature immune synapse upon antigen recognition on antigen presenting cells (APCs), as well as induction of IL-2 production and T cell proliferation. In response to T cell costimulation, the actin-binding protein L-plastin (LPL) becomes activated by phosphorylation at its serine-5 residue and can subsequently fulfil its crucial function for immune synapse formation between T cells and APC, and the upregulation of the T cell activation markers CD25 and CD69 on the cell surface (32–34). Another costimulation-induced event required for T cell activation is the dephosphorylation of the actin-severing and depolymerizing protein cofilin at serine-3 (35–37). Since all Arnica extracts as well as thymol and helenalin inhibited the production of IL-2 and T cell proliferation, next their influence on the activation of the actin-binding proteins LPL and cofilin was investigated. Western Blot analysis of total T cell lysates prepared after 30 min CD3xCD28 stimulation revealed that neither the Arnica extracts (**Figures 6A, B, E**, **G**) nor thymol or helenalin (**Figures 6C, D, F** and **H**) had a significant effect on LPL phosphorylation and cofilin dephosphorylation in human PBTs.

### 3.7 Thymol and helenalin, but not Arnica extracts inhibit T cell migration

Apart from proliferation, cellular migration represents an important function of T cells, as it enables them to reach, for example, sites of infection or inflammation throughout the human body. To analyze whether Arnica extracts influence the migratory capacity of PBTs, a Transwell™ assay was performed. Thereby, cells are placed in the upper compartment that is separated by a semi-permeable membrane from the lower, chemoattractant-containing compartment. T cells that migrated towards the chemokine stromal cell-derived factor 1α (SDF-1α) contained in the lower chamber were quantified by flow cytometry. Arnica extracts did not affect PBT migration towards SDF-1α (**Figure 6I**). In contrast, the pure compounds thymol (500 μM) and helenalin (0.25 μM) significantly inhibited PBT migration (**Figure 6J**). As mentioned above, cell viability, as detected by 7-AAD staining, was not affected by these substances (**Supplementary Figure S5**). The finding, that Arnica extracts did not influence the migratory capacity of PBT, in addition, confirmed that the Arnica extracts did not affect T cell viability.

### 3.8 Arnica extracts, thymol and helenalin intervene differently in T cell receptor signaling and inhibit cytokine production

**Figure 7:**
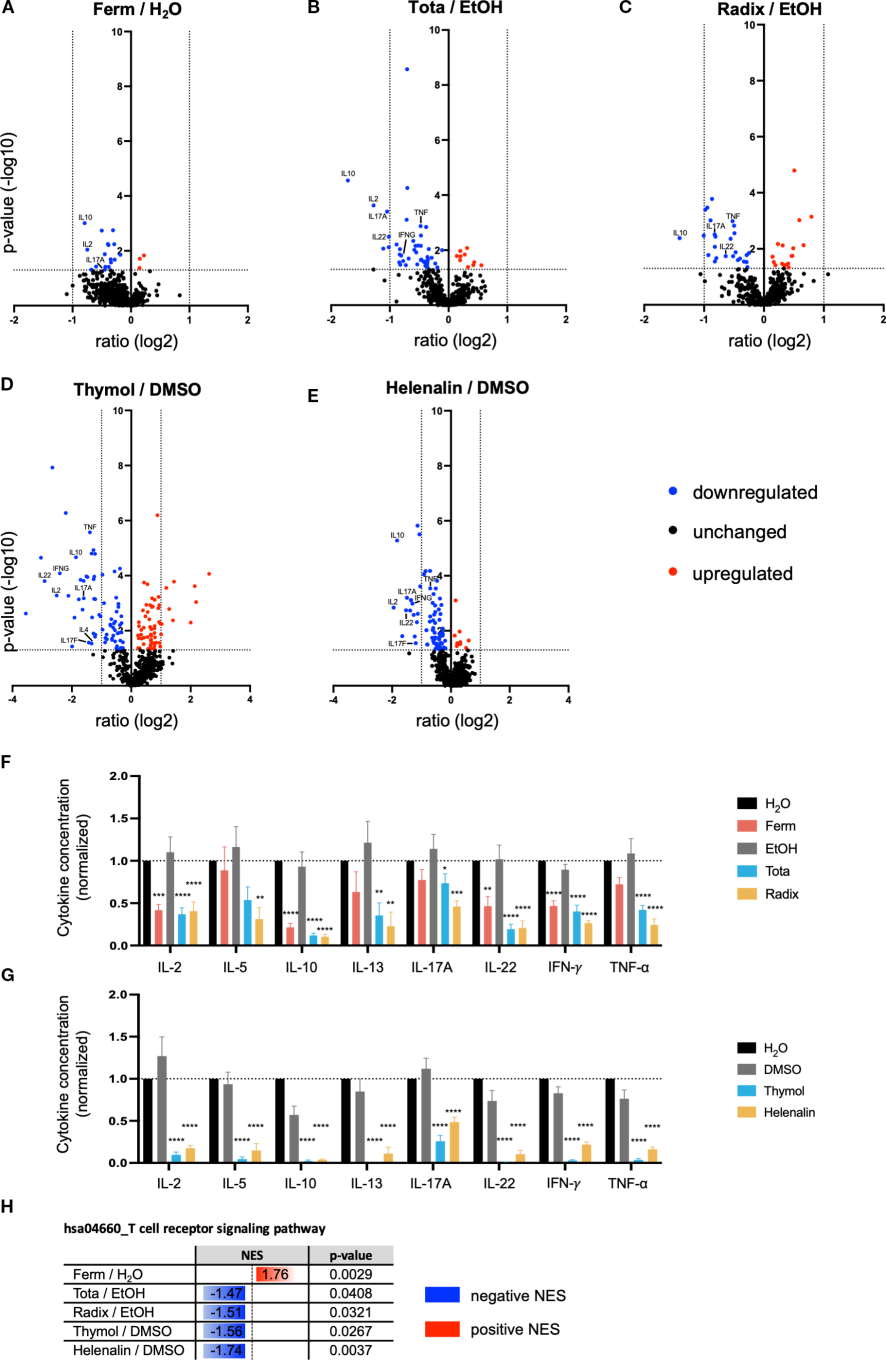
Arnica extracts, thymol and helenalin intervene differently in T cell receptor signaling and inhibit cytokine production. **(A–E)** PBTs were pretreated with drug or vehicle for 1 h and subsequently activated with anti–CD3/CD28 antibodies for 4 h. Total RNA was isolated from five independent experiments and mRNA transcripts of 579 immune–related genes (Human Immunology V2 Panel) were counted using the nCounter® technology by nanoString. Pairwise comparisons of gene expression from drug treated samples versus the respective solvent control are shown as volcano plots. Significantly downregulated (blue) and upregulated genes (red) are indicated. Some interesting candidates are labeled. **(F, G)** PBTs were pretreated with drug (red, blue, and yellow bars) or vehicle (black and grey bars) for 1 h and subsequently activated with anti-CD3/CD28 antibodies for 24 h. Different T helper cell-related cytokines (IL–5, IL–10, IL-13, IL-17A, IL-22, IFN-γ, and TNF-α) released to the supernatant after treatment with Arnica extracts **(F)** or pure compounds **(G)** were quantified by cytometric bead array (LEGENDplex™) using supernatants from six independent experiments. Data were normalized to the H_2_O control sample and are expressed as mean ± SEM. *p ≤ 0.05; **p ≤ 0.01; ***p ≤ 0.001; ****p ≤ 0.0001. **(H)** Gene expression data were used to perform a KEGG pathway gene set enrichment analysis. Normalized enrichment scores (NESs) and *p-*values of the different test substances compared to the respective solvent control for the T cell receptor signaling pathway (hsa04660) are shown.

To gain a broad understanding of the underlying molecular mechanism of drug action, gene expression analysis was performed using the nCounter^®^ platform. This technique allows the comparison of gene expression in different RNA samples by means of simultaneously counting individual mRNA molecules for the gene set of interest without prior amplification. Using the Human Immunology V2 panel covering 579 immune-related genes, gene expression in PBTs that were pretreated with different Arnica extracts (**Figures 7A–C**), thymol (**Figure 7D**), or helenalin (**Figure 7E**) and costimulated with CD3xCD28 was compared to the corresponding solvent control sample (H_2_O or EtOH, respectively). In the volcano plots (**Figures 7A–E**), genes that were significantly downregulated are marked in blue and genes that were significantly upregulated are marked in red. After treatment with Ferm (**Figure 7A**), Tota (**Figure 7B**) or helenalin (**Figure 7E**) the majority of significantly regulated genes were downregulated (marked in blue). Interestingly, Radix (**Figure 7C**) and thymol (**Figure 7D**) also showed a number of upregulated genes (marked in red). A summary of the significantly regulated genes is provided in **Supplementary Table S2**.

Evaluation of the nCounter® analysis, as shown in the volcano plots (**Figures 7A–E**), revealed that the expression of several cytokine genes was downregulated by all three extracts, as well as by thymol and by helenalin, but with different specificities. To study the production of these cytokines at the protein level, a LEGENDplex™ analysis of cell culture supernatants after 24 h stimulation was performed (**Figure 7F**). In addition to IL-2 (see also **Figure 3A**), Ferm, Tota and Radix extracts significantly decreased the levels of IL-10, IL-22, and IFN-γ. The cytokines, IL-13, IL-17A, and TNF-α were significantly decreased by the Tota and Radix extracts but not by the Ferm extract. Similar to Tota and Radix, thymol and helenalin significantly decreased the levels of all these cytokines in the cell culture supernatants (**Figure 7G**). IL-5 was only significantly reduced by Radix, thymol and helenalin. The levels of IL-4, IL-6, IL-9 and IL-17F were below the limit of quantification.

The KEGG TCR signaling pathway map shown in **Supplementary Figure S6** illustrates the components that link T cell receptor triggering to T cell effector functions, namely T cell proliferation, differentiation, and the production of various cytokines such as IL-2, IL-5, IL-10, IFN-γ, and TNF-α. A gene set enrichment analysis (GSEA) revealed that the KEGG TCR signaling pathway (hsa04660) is differentially targeted by the Arnica extracts (**Figure 7H**). While treatment with Tota or Radix extract resulted in a negative normalized enrichment score (NES), Ferm extract, surprisingly, showed a positive NES compared to the solvent control. Similar to Tota and Radix, helenalin and thymol resulted in a negative NES. These results, together with the reduced expression of cytokine genes and the reduced cytokine concentrations in the supernatant, support the hypothesis that the TCR signaling pathway represents a molecular target of Arnica extracts, thymol and helenalin in human PBTs.

### 3.9 Nuclear translocation of NFκB is inhibited by thymol, but not by Arnica extracts

**Figure 8:**
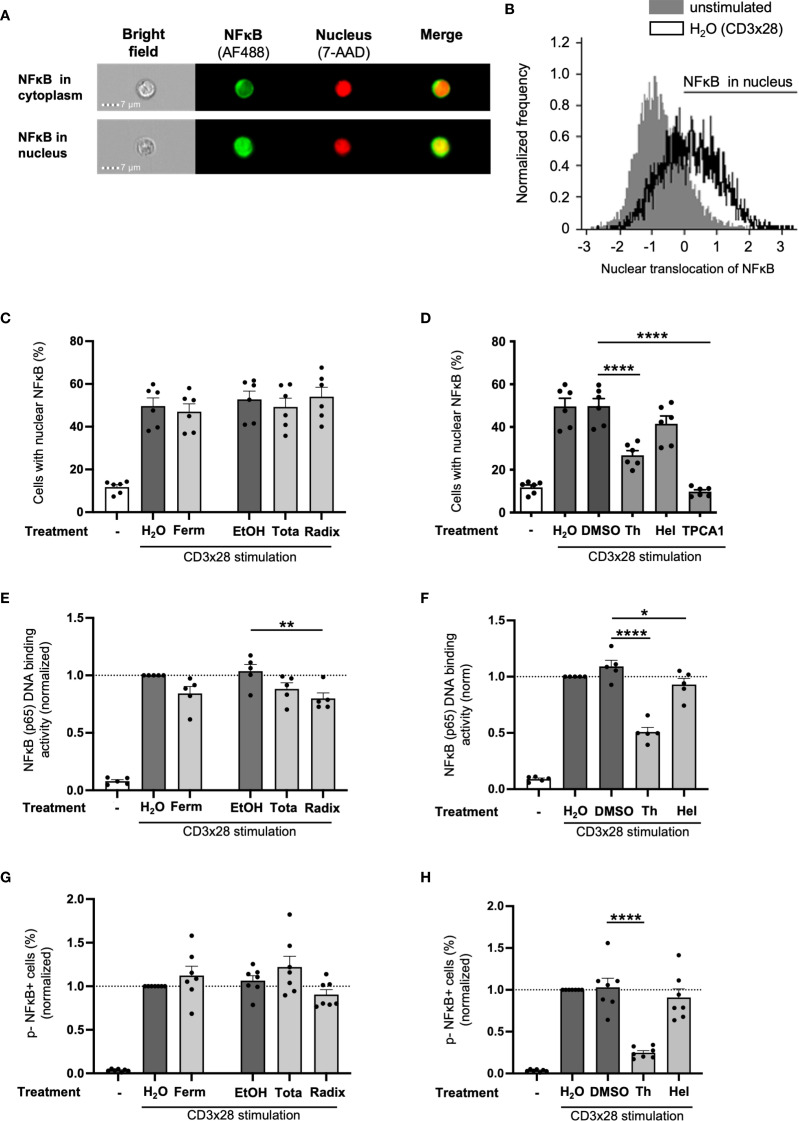
Differential effects of Arnica extracts, thymol and helenalin on the nuclear translocation, DNA-binding and phosphorylation of NFκB. PBTs were left unstimulated (left white bar) or pretreated with drug (light grey bars) or vehicle (dark grey bars) for 1 h and subsequently activated with anti-CD3/CD28 antibodies for 30 min. **(A)** Nuclear translocation of NFκB p50 was quantified by imaging flow cytometry. **(B)** Representative histograms of unstimulated versus costimulated H_2_O control sample. Statistical evaluation of NFκB nuclear translocation after treatment with Arnica extracts **(C)** or pure compounds or TPCA1 **(D)** from six independent experiments. Data are expressed as mean ± SEM. **(E, F)** The DNA binding activity of NFκB p65 in nuclear lysates was assessed by means of an ELISA-based detection method. Statistical evaluation of NFκB DNA binding after treatment with Arnica extracts **(E)** or pure compounds **(F)** from five independent experiments. **(G, H)** Statistical evaluation of NFκB p65 phosphorylation on S529 after treatment with Arnica extracts **(G)** or pure compounds **(H)** analyzed by flow cytometry in seven independent experiments. Each data point represents an individual T cell donor. Data were normalized to the H_2_O control sample and are expressed as mean ± SEM. *p ≤ 0.05; **p ≤ 0.01; ****p ≤ 0.0001.

Within the complex TCR signaling network, the transcription factor family NFκB is one of the key regulators of *IL2* expression. To further clarify the molecular mechanisms underlying diminished IL-2 production upon treatment with Arnica extracts, their effect on different steps within the NFκB signaling pathway was evaluated. In unstimulated cells, NFκB, consisting of the subunits p65 and p50, is bound to its inhibitor IκB and localized in the cytoplasm. NFκB needs to translocate to the nucleus to become transcriptionally active. CD3xCD28 costimulation of T cells results in phosphorylation and degradation of the inhibitor IκB. Thereby, NFκB is released and can subsequently enter the nucleus via its nuclear localization sequence (38). Analysis of NFκB nuclear translocation by imaging flow cytometry after costimulation of PBTs through CD3xCD28 (**Figures 8A, B**) revealed that Ferm, Tota, and Radix extract had no significant influence on the percentage of cells containing nuclear NFκB (**Figure 8C**). In contrast to Arnica extracts, the pure compound thymol, and the IκB kinase inhibitor TPCA1 (10 μM), which served as positive control, significantly reduced nuclear translocation of NFκB (**Figure 8D**). A cytotoxic effect of TPCA1 treatment was excluded by 7-AAD staining (**Supplementary Figure S7A**). Helenalin inhibited the nuclear translocation of NFκB by trend (**Figure 8D**).

### 3.10 Arnica Radix extract specifically inhibits NFκB DNA binding

Within the nucleus, NFκB will bind to its DNA response element in the promoter region of target genes to initiate gene expression. The DNA binding capacity of NFκB in nuclear lysates of PBTs treated with test drug or vehicle was assessed using an ELISA-based assay. Therefore, wells coated with a consensus double-stranded DNA (dsDNA) sequence were incubated with nuclear lysate and DNA-bound NFκB was labeled with antibodies and a colorimetric readout was performed. Interestingly, only the Radix extract significantly reduced NFκB (p65) DNA binding. Although Ferm and Tota extracts inhibited IL-2 production to a similar extent, they only reduced NFκB DNA binding by trend (**Figure 8E**). In line with these data, thymol (mainly present in Radix) also reduced the amount of NFκB bound to DNA, while helenalin (mainly present in the upper plant parts) had only a small effect (**Figure 8F**).

Besides binding to DNA, transcriptional activation of NFκB is required to induce IL-2 gene transcription. The transcriptional activity of the NFκB p65/p50 heterodimer is regulated by different phosphorylation sites within the p65 subunit. Phosphorylation at Ser529 was described to promote transcriptional activity (39). Therefore, phosphorylation of the p65 subunit at Ser529 was analyzed by flow cytometry and used as a readout to study if Arnica extracts affect the NFκB pathway at this regulatory level. However, none of the extracts significantly influenced the percentage of phospho- NFκB ^+^ PBTs (**Figures 8G**, **Supplementary Figure S7C**). Only thymol significantly interfered with NFκB (p65) Ser529 phosphorylation, whereas helenalin treatment had no effect (**Figure 8H**, **Supplementary Figure S7D**).

### 3.11 Arnica (Tota) Ferm extract reduces NFAT-dependent gene expression through a calcium-influx independent mechanism

**Figure 9:**
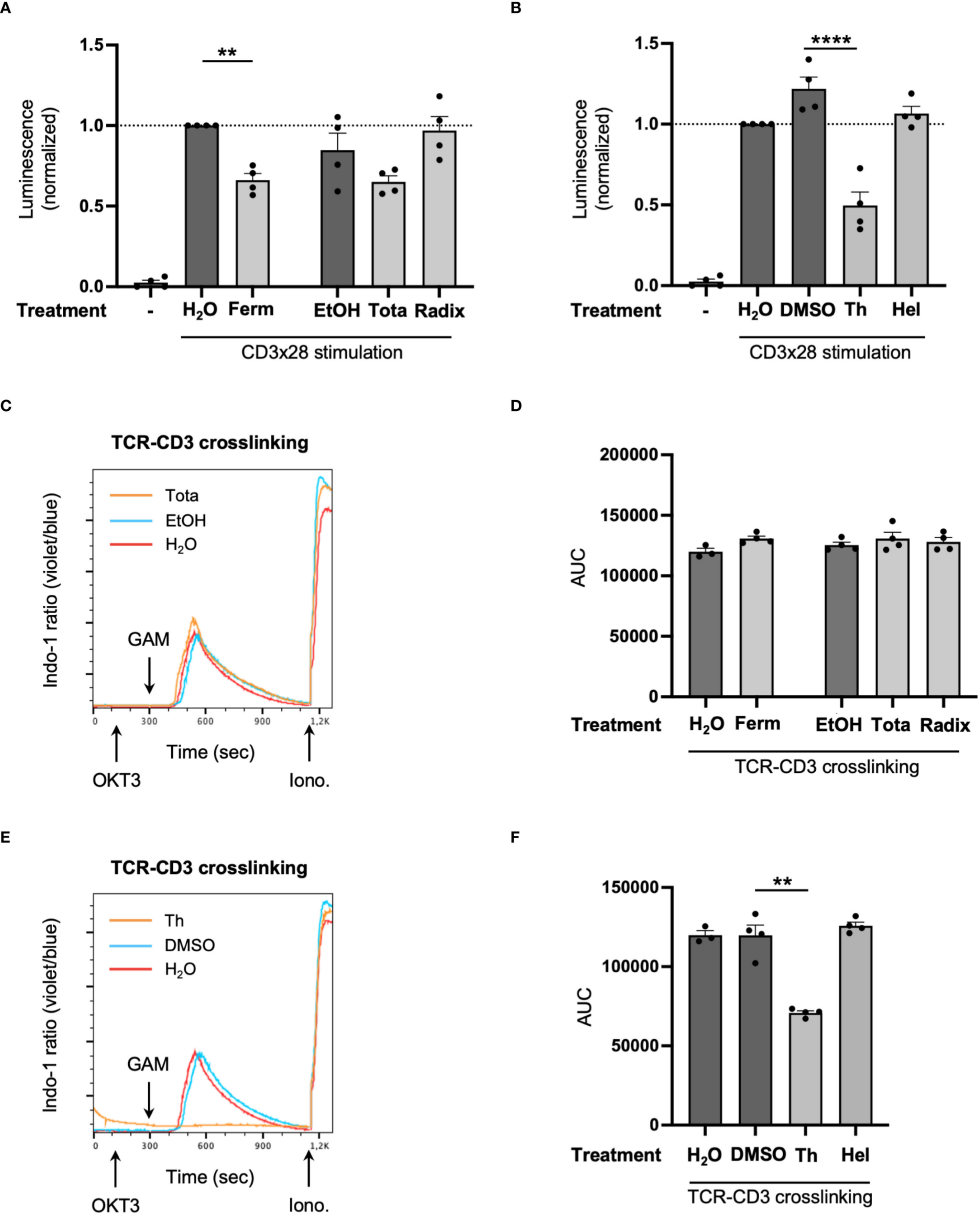
Arnica Ferm extract reduces NFAT-dependent gene expression through a calcium-influx independent mechanism. **(A, B)** Jurkat E6.1 cells were transfected with NFAT-pGL2-Promoter vector reporter plasmid and either left unstimulated (left white bar) or pretreated with drug (light grey bars) or vehicle (dark grey bars) for 1 h and subsequently activated with anti-CD3/CD28 antibodies for 6 **(H)** NFAT dependent gene expression after treatment with Arnica extracts **(A)** or pure compounds **(B)** was quantified by measuring luciferase activity in four independent experiments. Each data point represents an individual T cell donor. Data were normalized to the H_2_O control sample. **(C–F)** PBTs were labeled with Indo-1, pretreated with drug (light grey bars) or vehicle (dark grey bars) for 1 h and subsequently activated by TCR-CD3 crosslinking. The effect of Arnica extracts **(C, D)** or pure compounds **(E, F)** on stimulation-induced calcium influx was analyzed by flow cytometry. Representative kinetics of intracellular calcium levels after treatment with Arnica Tota extract **(C)** or thymol **(E)** compared to the respective solvent control and statistical evaluation of four independent experiments **(D, F)**. Each data point represents an individual T cell donor. Data are expressed as mean ± SEM. **p ≤ 0.01; ****p ≤ 0.0001.

Apart from NFκB, the NFAT transcription factor family is critically involved in controlling *IL2* expression. Similar to NFκB, NFAT is retained in the cytoplasm of unstimulated T cells. Upon activation it becomes dephosphorylated and enters the nucleus to initiate target gene transcription (40). To study NFAT-dependent gene expression and the effect of Arnica extracts, a luciferase reporter assay was performed. Since transfection of PBTs proved difficult, the Jurkat T leukemia cell line was used for this assay. A cytotoxic effect of the test drugs on this cell type was excluded beforehand (**Supplementary Figure S8A**). Firefly luciferase activity, measured by means of luminescence, as a read out for NFAT-dependent gene expression was significantly decreased upon treatment with Ferm extract (**Figure 9A**). Tota extract treatment resulted in a slight reduction, which did not reach statistical significance. For Radix extract, an effect on NFAT-dependent gene expression could be excluded, underlining its specific action on NFκB signaling (compare **Figure 8**).

NFAT activation through dephosphorylation is mediated by calcium-dependent activation of the serine phosphatase calcineurin. Therefore, stimulation-induced Ca^2+^ influx is one factor regulating NFAT activation. To study the effect of Arnica extracts on intracellular Ca^2+^ levels, PBTs were stimulated by TCR-CD3 crosslinking and Ca^2+^ influx was measured using the Indo–1 Ca^2+^ sensor. As expected, TCR engagement nicely increased intracellular Ca^2+^ levels (**Figures 9C–F**). Since none of the extracts had a significant influence on the Ca^2+^ influx compared to solvent control (**Figure 9D**), Arnica Ferm obviously decreased NFAT-dependent gene expression by a calcium-independent mechanism or at least downstream of the Ca^2+^ rise. Interestingly, in line with findings in other cell types (41–43), thymol even increased Ca^2+^ in resting PBTs (**Supplementary Figures S8B, C**). However, it significantly diminished the Ca^2+^ influx in CD3xCD28 stimulated PBTs (**Figures 9E, F**) and reduced NFAT-dependent gene expression (**Figure 9B**). In contrast, helenalin had no effect on these two parameters (**Figures 9B, F**).

## 4 Discussion

*Arnica montana* L. (Arnica) is traditionally applied to inhibit inflammation and promote healing of blunt injuries like contusions or bruises. CD4^+^ T cells play an important role in the normal muscle healing process in response to trauma. However, persistence of activated CD4^+^ and CD8^+^ T cells results in sustained inflammation and delayed muscle repair (14). In this study, we evaluated the immunomodulatory capacity of Arnica extracts on primary human T cells to allow a critical evaluation of Arnica’s mode of action based on consolidated experimental data. Our approach of a side-by-side comparison of three differentially manufactured extract candidates offers the possibility to gain a deeper understanding of how, for example, the used plant part or the extraction solvent influence their cellular effects.

In our study, we preferentially used primary human PBTs activated with anti-CD3/CD28 antibodies, which mimics the physiological situation, namely the costimulation of T cells by professional APCs. Although working with cell lines such as Jurkat T leukemia cells (2) or the J774 murine macrophage cell line (44), has technical advantages, including easy availability and handling, results obtained exclusively on cell lines cannot be directly translated to primary cells due to possible differences, e.g. in intracellular signal transduction (45, 46). Using the experimental system described above, an inhibitory effect of all tested Arnica extracts on CD25 surface expression and T cell proliferation was identified, which was not described before. Arnica treatment also inhibited production of the growth factor IL-2. However, the lack of IL-2 is not solely responsible for the decreased cell division rate, as IL-2 supplementation could not restore normal proliferation. In conclusion, it is a combination of diminished IL-2 production and decreased affinity of the IL-2R resulting from reduced IL-2Rα chain (CD25) expression that can explain the inhibitory effect of Arnica on T cell proliferation. Apart from IL-2, Arnica-treated PBTs released, inter alia, less IL-10, IFN-γ, and TNF-α. A decrease in TNF-α production upon treatment with Arnica flower tincture was also found for LPS-stimulated peripheral blood mononuclear cells (PBMCs) that contain lymphocytes and monocytes (2). Similarly, in the presence of hydroalcoholic whole plant Arnica extract, the human monocytic cell line THP-1 produced less TNF–α in response to LPS and IFN-γ stimulation (47).

Fitting to hampered cytokine production and cell division in PBTs, nCounter® gene expression analysis followed by gene set enrichment analysis revealed that Arnica preparations intervene in TCR signal transduction cascades. Transcription of *IL2* and other cytokine genes following T cell activation is controlled by several transcription factor families, including NFκB and NFAT. Interestingly, despite the fact that the three Arnica extracts all inhibited IL-2 production to a similar extent, they differentially affected T cell signaling pathways leading to IL-2 expression (summarized in **Table 3**). The hydroethanolic root extract (Radix) selectively inhibited NFκB DNA binding, while the aqueous fermented extract (Ferm), for which the whole fresh flowering plant was used, predominantly suppressed NFAT-dependent gene expression. The hydroethanolic whole plant extract (Tota) exerted a moderate effect on both pathways, yet without reaching statistical significance. An effect on NFAT DNA binding, was previously described for Arnica tinctures containing mainly sesquiterpene lactones (2). Thereby, NFAT was completely inhibited by lower concentrations. This suggests that the effect on NFAT described here may be attributable to the helenalin and dihydrohelenalin derivatives detected in the aqueous fermented extract and in lower amounts in the hydroethanolic whole plant extract. *Klaas et al.* (2) showed additionally, that the esterification of the sesquiterpene lactones plays an important role in their activity. That may explain, why the pure compound helenalin showed no effect on NFAT-dependent gene expression in the present study.

**Table 3:** Differential effects of Arnica extracts, thymol and helenalin on NFkB versus NFAT signaling.

	Proliferation	IL-2 production	NFκB nuclear translocation	NFκB DNA binding	NFAT-dependent gene expression
Ferm	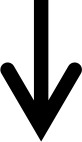	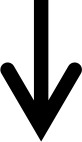	**-**	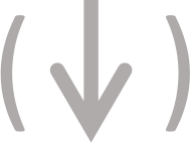	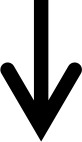
Tota	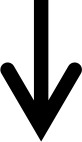	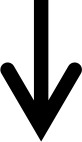	**-**	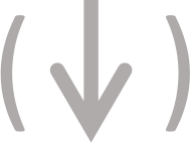	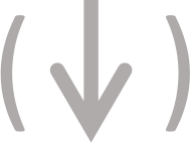
Radix	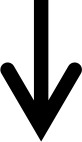	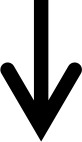	**-**	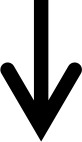	**-**
Thymol	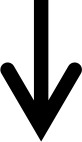	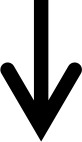	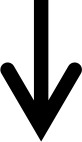	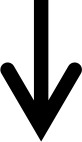	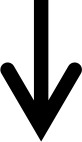
Helenalin	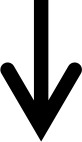	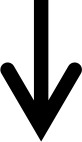	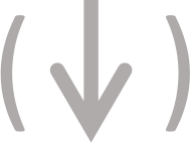	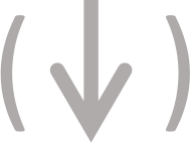	**-**

Dilutions and concentrations used were: Ferm (1:1000), Tota (1:1000), Radix (1:1000), helenalin (0.25 μM) and thymol (500 μM). Dark arrows mean a significant effect, light arrows show effects by trend.

In line with the results for NFκB DNA binding, *Röhrl et al.* recently described that a hydroethanolic whole plant extract of Arnica inhibited NFκB reporter expression in calcium ionophore and PMA stimulated Jurkat T leukemia cells (48). An inhibitory effect on the nuclear translocation of NFκB, as described for the LPS–stimulated murine macrophage cell line J774 treated with a methanolic whole plant extract (44), was, however, not detected in our experimental system using CD3xCD28 costimulated primary human T cells treated with the hydroethanolic whole plant extract (Tota). Several factors, including differences in the cell type used (macrophage cell line versus primary PBTs), stimulation condition (LPS versus anti-CD3/CD28 stimulation), and the plant extraction solvent (methanol versus 62% ethanol), may explain this discrepancy.

Importantly, the Radix extract prepared from the dried roots by hydroethanolic extraction specifically interfered with NFκB DNA binding and efficiently inhibited T cell activation and proliferation. Consistent with these findings, thymol, mainly present in Radix, also decreased NFκB DNA binding, whereas inhibition by helenalin occurred to a lesser extent. These findings were particularly interesting, since pure Arnica root extracts were not systematically studied for their immunomodulatory potential so far. This is probably related to the assumed lack of bioactive SLs that are enriched specifically in the flower heads. Therefore, future studies on Arnica should no longer be limited to flower-derived or whole plant extracts, and the plant parts contained in the test material with their specific composition should be clearly differentiated.

Similar to the Arnica extracts, also thymol and helenalin diminished CD25 expression, IL-2 production and proliferation of PBTs. So far, the effect of thymol on the activation of isolated primary human T cells has not been investigated. Only for total peripheral blood lymphocytes (PBLs) and Jurkat T leukemia cells less proliferation upon thymol treatment has been reported (49). In another study using Jurkat T leukemia cells thymol treatment also reduced IL-2 production (50). Our results show that thymol inhibited nuclear translocation of NFκB/NFκB DNA binding, and phosphorylation of the NFκB p65 subunit at S529 in PBTs. Thymol also interfered with calcium signaling and NFAT-dependent gene expression. Diminished nuclear translocation of NFAT2 was found in thymol-treated Jurkat T leukemia cells (50), supporting our finding that thymol decreased NFAT-dependent gene expression. Divergent data were described for phosphorylation of the NFκB p65 subunit. While thymol diminished S529 phosphorylation in PBTs (our present study), *Gholijani et al.* found no significant influence in Jurkat cells, yet without specifying the phosphorylation site (50). Thus, differences in untransformed human PBTs versus transformed Jurkat leukemia cells, potential differences in the analyzed phosphorylation site and the use of nuclear extracts for Western Blotting (*Gholijani et al.*) versus flow cytometric analysis in the entire cell (present study) impair direct comparability. Using LPS-stimulated RAW264.7 murine macrophage cells, Thymol proved to be anti-inflammatory, inter alia, by inhibiting TLR4-mediated NFκB p65 subunit phosphorylation (51). Although these data fit to the results described for NFκB p65 S529 phosphorylation in PBTs in the present study, again, a direct comparison is not possible due to missing information on the specific phosphorylation site investigated by *Wu et al.* (51).

While it was known from studies using non-immune cells that thymol treatment induces spontaneous calcium influx (41–43), this effect was not yet described for primary T cells. Also, the inhibition of stimulation-induced calcium influx in primary human PBTs (our present study), provides novel insights into the molecular mechanism underlying thymol’s effects. An inhibition of calcium mobilization by thymol was observed before for N-formyl-methionyl-leucyl-phenylalanine (fMLP)-stimulated neutrophils (52). Due to its hydrophobic nature, thymol can easily cross cell membranes and was found to act on various ion channels, including members of the TRP family calcium channels (53–55). The differential influence of thymol on calcium influx in primary resting versus activated T cells is a novel and intriguing finding. Yet, the underlying molecular mechanism remains to be elucidated. It is conceivable that thymol may interfere with calcium entry in T cells by affecting store-operated calcium channels such as ORAI1, or by altering membrane fluidity and indirectly modulating channel function. Future studies will be required to clarify these potential mechanisms.

Helenalin inhibited CD25 expression, IL-2 production and proliferation of human PBTs. In line with these data, *Berges et al.* found that helenalin inhibited CD25 expression, IL-2 production and proliferation of CD4^+^ PBTs (3). However, those results cannot be directly compared, as *Berges et al.* focused on the apoptosis-inducing action of helenalin and worked with an, at least in our hands, toxic drug concentration. Accordingly, in our study no effect of helenalin on NFAT-dependent gene expression in PBT was observed. In contrast, *Berges et al.* found significantly diminished nuclear translocation of NFAT1 in CD4^+^ PBTs in response to helenalin (3). Again, this finding might be related to the use of a cytotoxic drug concentration, whereas a lower and certainly non-toxic concentration was used in our present study.

Helenalin inhibited the nuclear translocation of NFκB in human PBT by trend and showed a small but significant inhibition of NFκB DNA binding. Note that the use of nuclear extracts from helenalin treated versus untreated cells in our DNA binding assay did not allow to distinguish whether less NFκB bound to DNA was only due to reduced DNA binding capacity or, in addition, resulting from slightly impaired nuclear translocation. Future studies employing techniques such as electrophoretic mobility shift assays (EMSA) or imaging-based approaches could help resolve this question and further clarify the mechanism of NFκB modulation. For Jurkat cells *Lyss et al.* (5) described that helenalin selectively alkylates cysteine sulfhydryl groups in the p65 subunit and thereby inhibits NFκB DNA binding. Thus, our results agree with *Lyss et al.*, although the helenalin concentrations were much higher in their study (10-20µM).

Synergistic or antagonistic interactions among compounds within the complex mixtures could contribute to these differential effects. Expectedly, and fitting to the observed differences in the molecular mode of action, phytochemical characterization of the extracts by LC-MS/MS and tentative identification revealed a completely different composition of the tested extracts. Not surprisingly, thymol was preferentially present in Radix, whereas helenalin was mainly found in whole plant extracts. Significant phytochemical differences between extracts prepared from the fresh versus dried whole plant were described also by Duthen et al. (22). Our tentative identification of phytochemical compounds based on literature revealed that caffeoylquinic acids make up the most prominent substance group in Tota and Radix. Therefore, we went on with testing pure mono-, di- and tri- caffeoylquinic acid in our system to see, if the effects of the Arnica extracts on T cell activation could be mimicked by these substances. However, although caffeoylquinic acids are among the most abundant constituents in Arnica extracts, they did not reproduce the immunosuppressive effects observed with the full extracts in our assays. This suggests that these compounds are either inactive in the tested context or may require metabolic transformation, cellular uptake modifications, or synergistic interactions with other extract components to become functionally active. Elucidating these possibilities will require further studies focused on compound stability, bioavailability, and potential synergistic effects within the plant extracts. While the current study does not dissect all possible interactions, our analysis of individual compounds provides some insight into their specific contributions.

In summary, this study elucidates the distinct immunomodulatory effects of three differently produced Arnica extracts on primary human T cells. Notably, the root extract (Radix) selectively inhibited NFκB DNA binding, while the fermented whole flower extract (Ferm) suppressed NFAT-dependent gene transcription (summarized in **Table 3**). These findings highlight the potential of Arnica extracts as anti-inflammatory agents targeting different T cell signaling pathways. Given their selective modulation of key transcription factors, these extracts may offer innovative therapeutic avenues for treating T cell-driven inflammatory conditions such as psoriasis and arthritis. Future research should focus on their effects on distinct disease-related T cell subpopulations and employ *in vivo* models to further delineate their - potentially different - therapeutic efficacy in chronic inflammatory diseases. Understanding their molecular mode of action will also facilitate evaluation of the potential benefits and risks of combining the different Arnica extracts with conventional drugs, such as cyclosporine A or FK506, which also inhibit NFAT signaling.

In the context of delayed muscle repair, associated with excess inflammation and prolonged presence of CD4+ and CD8+ T cells (14), inhibition of T cell activity by Arnica can indeed be beneficial to stop inflammation and to return to the normal muscle healing process. This notion is further supported by data describing that thymol promotes the differentiation of T cells towards immunosuppressive regulatory T cells (Tregs) (56). However, care has to be taken for the correct time point of interference. Especially CD4+ T cells are involved in initial creation of a pro–inflammatory milieu that is an essential part of the normal healing process (10, 11). Therefore, interfering with T cell activity too early may actually be detrimental rather than supportive of tissue repair, whereas later treatment may help to resolve overwhelming inflammatory activity and to prevent chronic inflammation. Overall, a detailed understanding of the spatio-temporal involvement of different immune cell types in muscle healing and chronic inflammation would be desirable to allow therapeutic intervention based on a precise knowledge of the cellular and molecular mechanisms that need to be addressed by Arnica at different times during the healing process.

Although our findings highlight the immunomodulatory potential of Arnica extracts *in vitro*, the absence of *in vivo* data represents a limitation of the current study. Animal models for T cell-mediated skin inflammation, such as psoriasis-like dermatitis, as well as models of impaired muscle regeneration following injury, would be suitable for assessing the precise immunomodulatory and tissue-regenerative potential of Arnica extracts, especially given its traditional use in musculoskeletal conditions. Such *in vivo* studies will be critical to evaluate the optimal treatment window and the reversibility of Arnica´s effects to guide future clinical translation.

## Data Availability

The datasets presented in this study can be found in online repositories. The names of the repository/repositories and accession number(s) can be found below: https://www.ncbi.nlm.nih.gov/geo/, GSE299224 https://www.ebi.ac.uk/metabolights/, MTBLS11351.
