# Correction: Distinct *Arnica montana* L. extracts modulate human T cell activation in different ways via differential inhibition of NFκB and NFAT pathways

**DOI:** 10.3389/fimmu.2025.1761559

**Published:** 2026-01-02

**Authors:** Karina M. Berschneider, Bernhard Wetterauer, Carsten Sticht, Christian Orlik, Beate Jahraus, Henning Kirchgessner, Anastasiia Zuieva, Stefan Wölfl, Divya Lairikyengbam, Verena Beier, Guido Wabnitz, Pille Wetterauer, Michael Schmiech, Yvonne Samstag

**Affiliations:** 1 Section Molecular Immunology, Institute of Immunology, Heidelberg University Hospital, Heidelberg, Germany; 2 Institute of Pharmacy and Molecular Biotechnology, Heidelberg University, Heidelberg, Germany; 3 Next Generation Sequencing Core Facility, Medical Faculty Mannheim, Heidelberg University, Mannheim, Germany; 4 Institute of Experimental and Clinical Pharmacology, Toxicology and Pharmacology of Natural Products, University of Ulm, Ulm, Germany

**Keywords:** *Arnica montana* L. extracts, primary human T cells (PBTs), immunomodulation, NFκB, NFAT, thymol, helenalin, LC-MS/MS

There was a mistake in **Table 3** as published. The arrow in the first row “Ferm”, last column “NFAT-dependent gene expression” must be dark grey and without brackets, showing a significant effect. The corrected **Table 3** appears below.

**Table 3:** Differential effects of *Arnica montana* L. extracts, thymol and helenalin on NFkB versus NFAT signaling.

	Proliferation	IL-2 production	NFκB nuclear translocation	NFκB DNA binding	NFAT-dependent gene expression
Ferm	**↓**	**↓**	**-**	(**↓**)	**↓**
Tota	**↓**	**↓**	**-**	(**↓**)	(**↓**)
Radix	**↓**	**↓**	**-**	**↓**	**-**
Thymol	**↓**	**↓**	**↓**	**↓**	**↓**
Helenalin	**↓**	**↓**	(**↓**)	(**↓**)	**-**

Dilutions and concentrations used were: Ferm (1:1000), Tota (1:1000), Radix (1:1000), helenalin (0.25 mM) and thymol (500 mM). Dark arrows mean a significant effect, light arrows show effects by trend.

The original version of this article has been updated.

