# Supplementary material for: Distinct Arnica montana L. extracts modulate human T cell activation in different ways via differential inhibition of NFκB and NFAT pathways
Source: Front Immunol. 2025 Oct 15;16:1655212. doi: 10.3389/fimmu.2025.1655212 (PMC12568507; doi:10.3389/fimmu.2025.1655212)
Supplement: Supplementary file 1 [file DataSheet1.zip › BerschneiderK_ArnicaExtracts_SupplementaryFigures.pdf]

Suppl. Figure 1

A

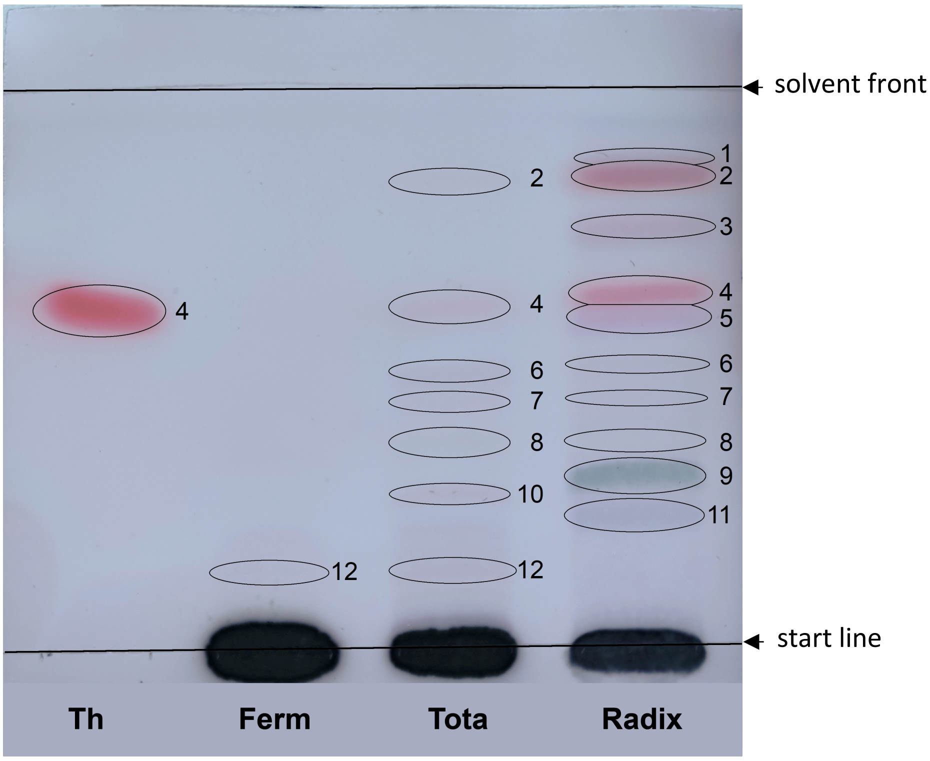

B

Nr.	R_f-values				Color	Tentative identification
	Thymol	Ferm	Tota	Radix		
1				0.88	gray	
2			0.84	0.83	russet	4–hydroxy–thymol–dimethyl ether [1]
3				0.75	grayish red	
4	0.61		0.61	0.63	red	Thymol
5				0.58	violet	Ethyl-phenylester [1, 2]
6			0.49	0.51	grey	
7			0.43	0.47	purple	
8			0.38	0.37	yellow	
9				0.29	green	Dienetriynes [2]
10			0.27		purple	
11				0.23	blue	
12		0.14	0.14		purple	

Suppl. Figure 1. TLC results of the examined commercial Arnica extracts.

(A) TLC plate after development with cyclohexane:diethylether (70:30) and reaction with anisaldehyde reagent. (**B**) R_f-values of selected bands and tentative identification [1,2].

[1] Brunner H. Dünnschichtchromatographie zur Prüfung von Arzneidrogen. 1. Mitteilung: Arnikawurzel. Deutsche Apotheker-Zeitung. 1969;109(44):1723–6.

[2] Rossetti V, Lombard A, Sancin P, Buffa M, Stefano RD. Characterization of *Arnica montana* L. dried roots. International Journal of Crude Drug Research. 1984;22(2):53–60. doi: 10.3109/13880208409070652.

Suppl. Figure 2

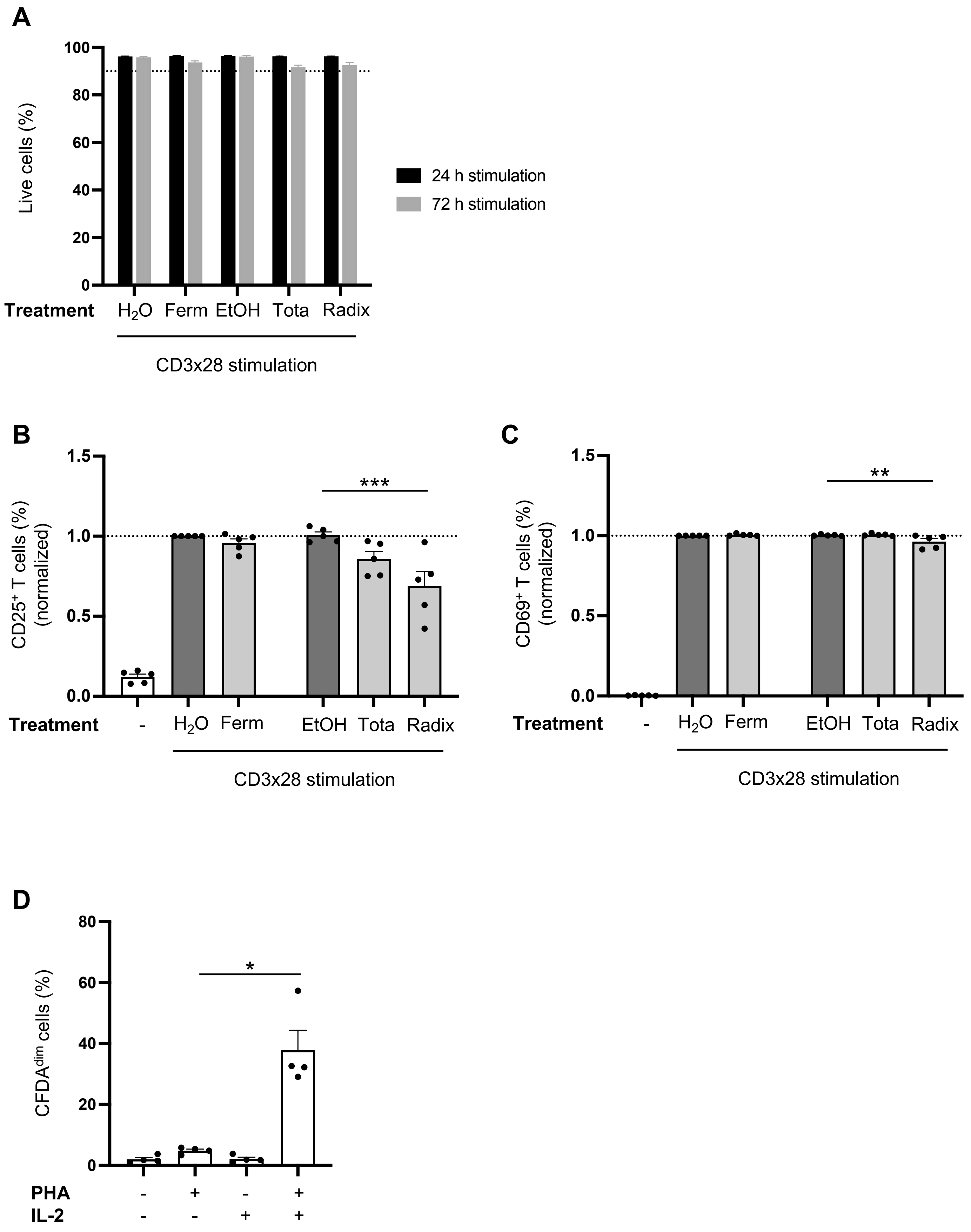

Suppl. Figure 2.

(A-C) PBT viability and activation marker expression after treatment with different Arnica extracts.

(**A**) PBTs were pretreated with Arnica extract (1:1000) or vehicle for 1 h and subsequently activated with anti-CD3/CD28 antibodies. Cell viability was assessed by 7-AAD exclusion after 24 h (black) and 72 h stimulation (grey). Data from five independent experiments are summarized and expressed as mean ± SEM. The threshold for non-toxic drug concentrations was set at 90% live cells. (**B**, **C**) PBTs were left unstimulated (white bar) or pretreated with Arnica extract (1:1000, light grey bars) or vehicle (dark grey bars) for 1 h and subsequently activated with anti-CD3/CD28 antibodies for 24 h. Surface expression of CD25 and CD69 was analyzed by flow cytometry. Statistical evaluation of the percentage of CD25-expressing ($CD25^+$) (**B**) and CD69-expressing ($CD69^+$) (**C**) PBTs from five independent experiments. Each data point represents an individual T cell donor. Data were normalized to the H_2O control sample and are expressed as mean ± SEM. $^{**}p \leq 0.01$; $^{***}p \leq 0.001$.

(D) Control experiment to prove functionality of recombinant human IL-2.

PBTs were labeled with CFSE and left untreated or pretreated with PHA overnight. After PHA removal, PBTs were incubated for 72 h with or without 40 U/ml recombinant human IL-2. T cell proliferation was assessed by CFSE dilution (four independent experiments). Each data point represents an individual T cell donor. Data are expressed as mean ± SEM. $^{*}p \leq 0.05$.

Suppl. Figure 3

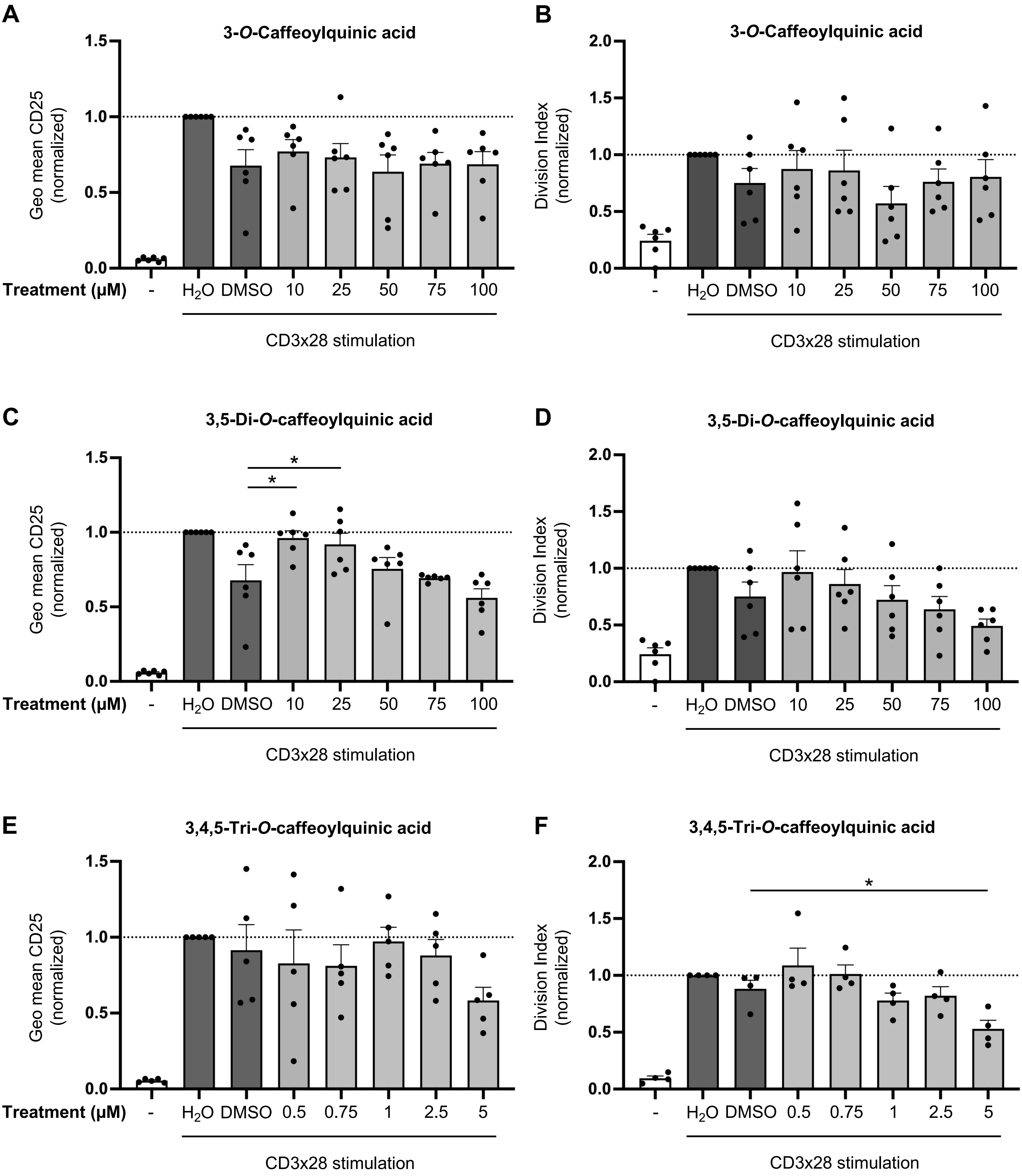

Suppl. Figure 3. Effects of Arnica extracts on CD25 expression and proliferation of human PBTs are not mimicked by pure (mono-, di-, or tri-) caffeoylquinic acid.

PBTs were left unstimulated (left white bar) or pretreated with substance as indicated (light grey bars) or vehicle (dark grey bar) for 1 h and subsequently activated with anti-CD3/CD28 antibodies. (**A**, **C**, **E**) Statistical evaluation of CD25 surface expression (Geo mean CD25) after 24 h stimulation analyzed by flow cytometry in 5–6 independent experiments. (**B**, **D**, **F**) Statistical evaluation of T cell proliferation after 72 h of stimulation assessed by CFSE dilution in 4–6 independent experiments. Data were normalized to the H_2O control sample. Each data point represents an individual T cell donor. Data are expressed as mean ± SEM. *p £ 0.05.

Suppl. Figure 4

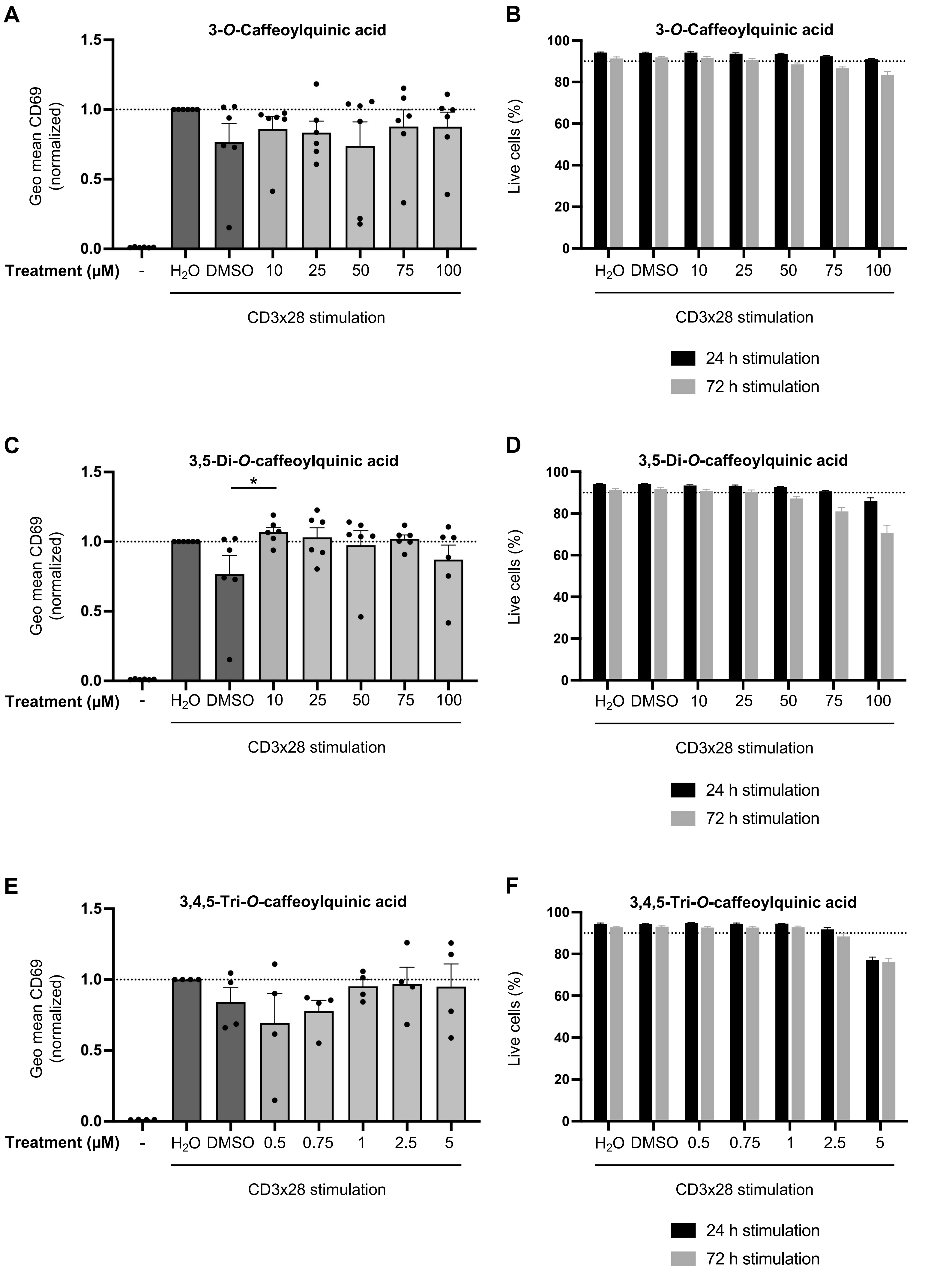

Suppl. Figure 4. CD69 surface expression and T cell viability after treatment with (mono-, di- or tri-) caffeoylquinic acid.

(**A**, **C**, **E**) PBTs were left unstimulated (left white bar) or pretreated with substance as indicated (light grey bars) or vehicle (dark grey bar) for 1 h and subsequently activated with anti-CD3/CD28 antibodies for 24 h. Statistical evaluation of CD69 surface expression on PBTs analyzed by flow cytometry in 4–6 independent experiments. Data were normalized to the H_2O control sample. Each data point represents an individual T cell donor. Data are expressed as mean ± SEM. *$p \leq 0.05$. (**B**, **D**, **F**) PBTs were pretreated with drug or vehicle for 1 h and then activated with anti-CD3/CD28 antibodies. Cell viability after 24 h (black) and 72 h of stimulation (grey) was assessed by 7-AAD exclusion in 5–6 independent experiments each. Data are expressed as mean ± SEM. The threshold for non-toxic drug concentrations was set at 90% live cells.

Suppl. Figure 5

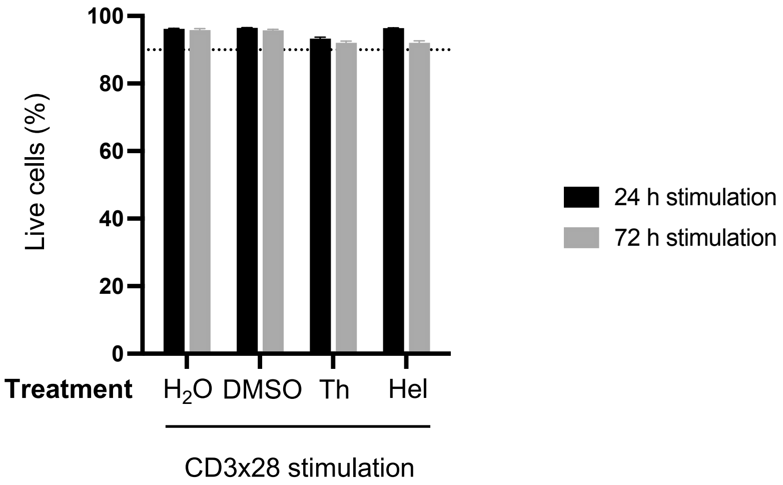

Suppl. Figure 5. Viability of PBTs after treatment with thymol or helenalin.

PBTs were pretreated with thymol (Th), helenalin (Hel) or vehicle for 1 h and subsequently activated with anti-CD3/CD28 antibodies. Cell viability was assessed by 7-AAD exclusion. Data from five independent experiments after 24 h (black) and 72 h stimulation (grey) are summarized. Data are expressed as mean ± SEM. The threshold for non-toxic drug concentrations was set at 90% live cells.

Suppl. Figure 6

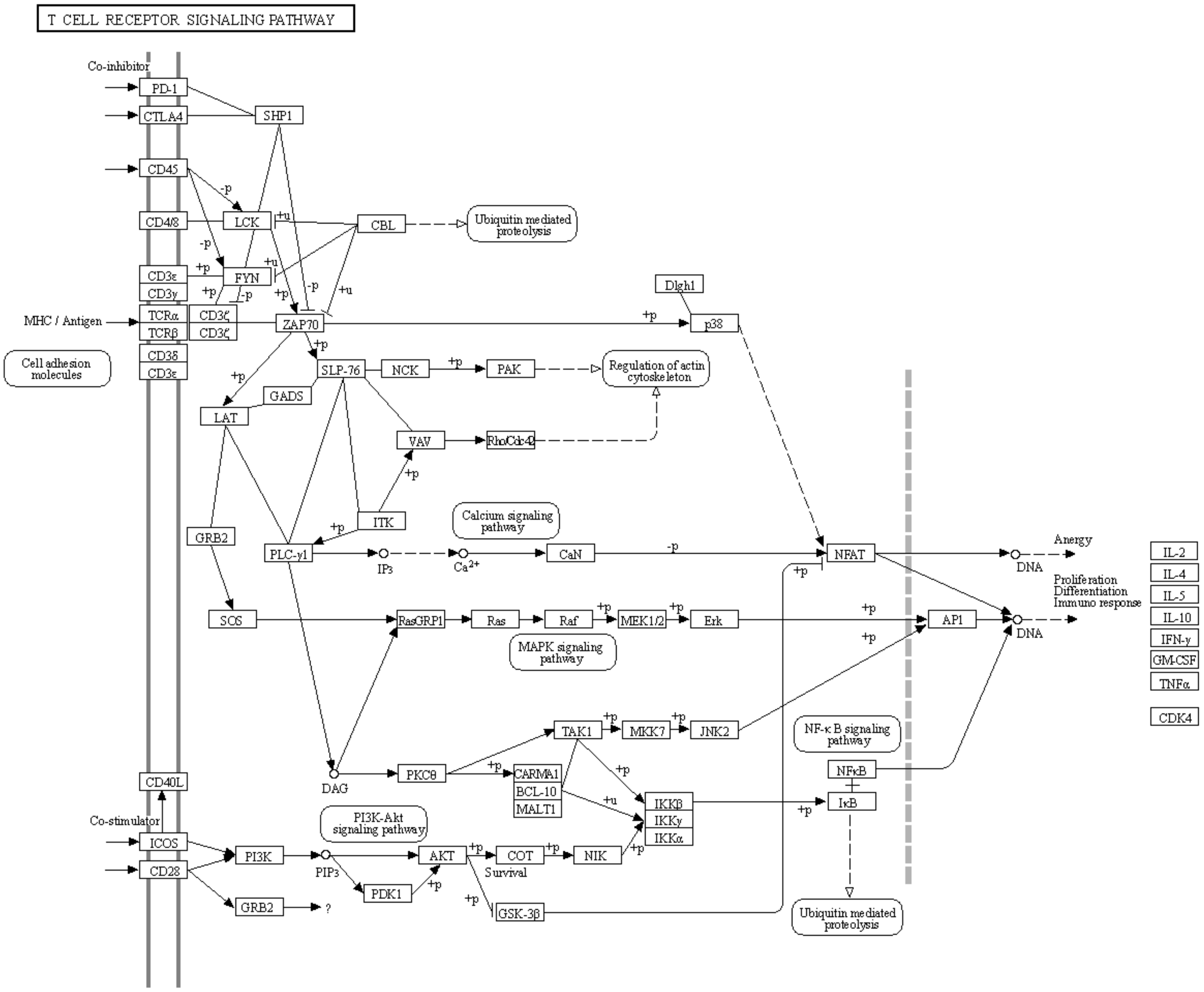

Suppl. Figure 6. KEGG T cell receptor signaling pathway map.
Schematic representation of the KEGG T cell receptor signaling pathway (https://www.genome.jp/pathway/hsa04660) [55].

Suppl. Figure 7

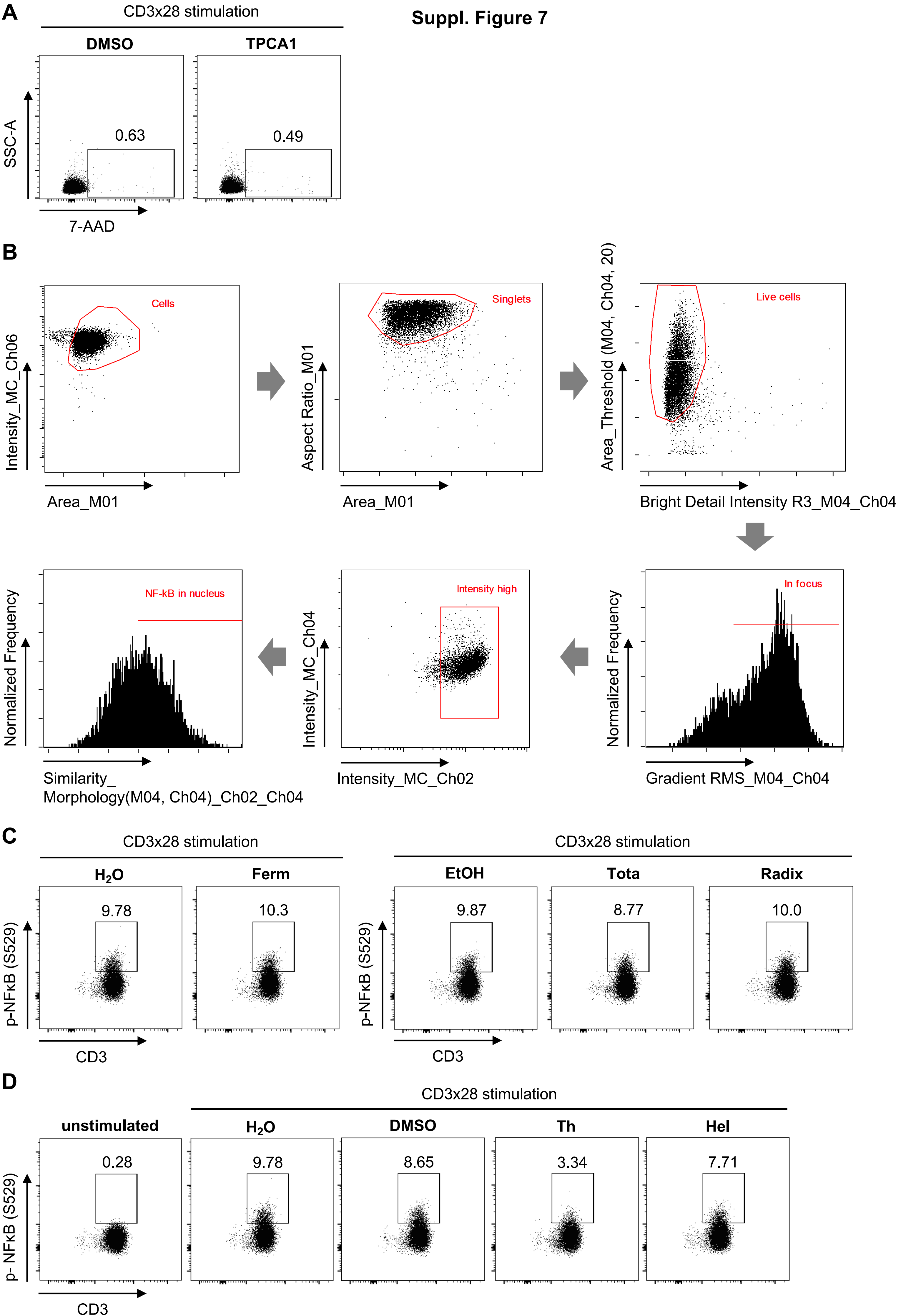

Suppl. Figure 7. PBT viability after TPCA1 treatment (A), gating strategy to study NFκBp50 nuclear translocation (B) and analysis of NFκBp65 phosphorylation at Ser529 in the presence or absence of Arnica extracts (C) or pure compounds (D).

(**A**) PBTs were pretreated with TPCA1 or DMSO for 1 h and subsequently activated with anti-CD3/CD28 antibodies for 1 h. Cell viability was assessed by 7-AAD exclusion. (**B**) Nuclear translocation of NFκB was analyzed by imaging flow cytometry. Sequential gating was performed to exclude cell doublets, apoptotic cells and cells out of focus from the analysis. For the remaining cells, a mask defining the cell nucleus was created based on the 7-AAD signal (Ch04). Nuclear translocation of NFκBp50, expressed as the percentage of cells with nuclear NFκB, was quantified by calculating the similarity between the cell nucleus mask and the NFκBp50 AF488 signal (Ch02). (**C**, **D**) PBTs were left unstimulated or pretreated with drug or vehicle (H_2O or EtOH) for 1 h and subsequently activated with anti-CD3/CD28 antibodies for 30 min. Phosphorylation of NFκBp65 on S529 after treatment with Arnica extracts (**C**) or pure compounds (**D**) was analyzed by flow cytometry. Representative dot plots from seven independent experiments are shown.

Suppl. Figure 8

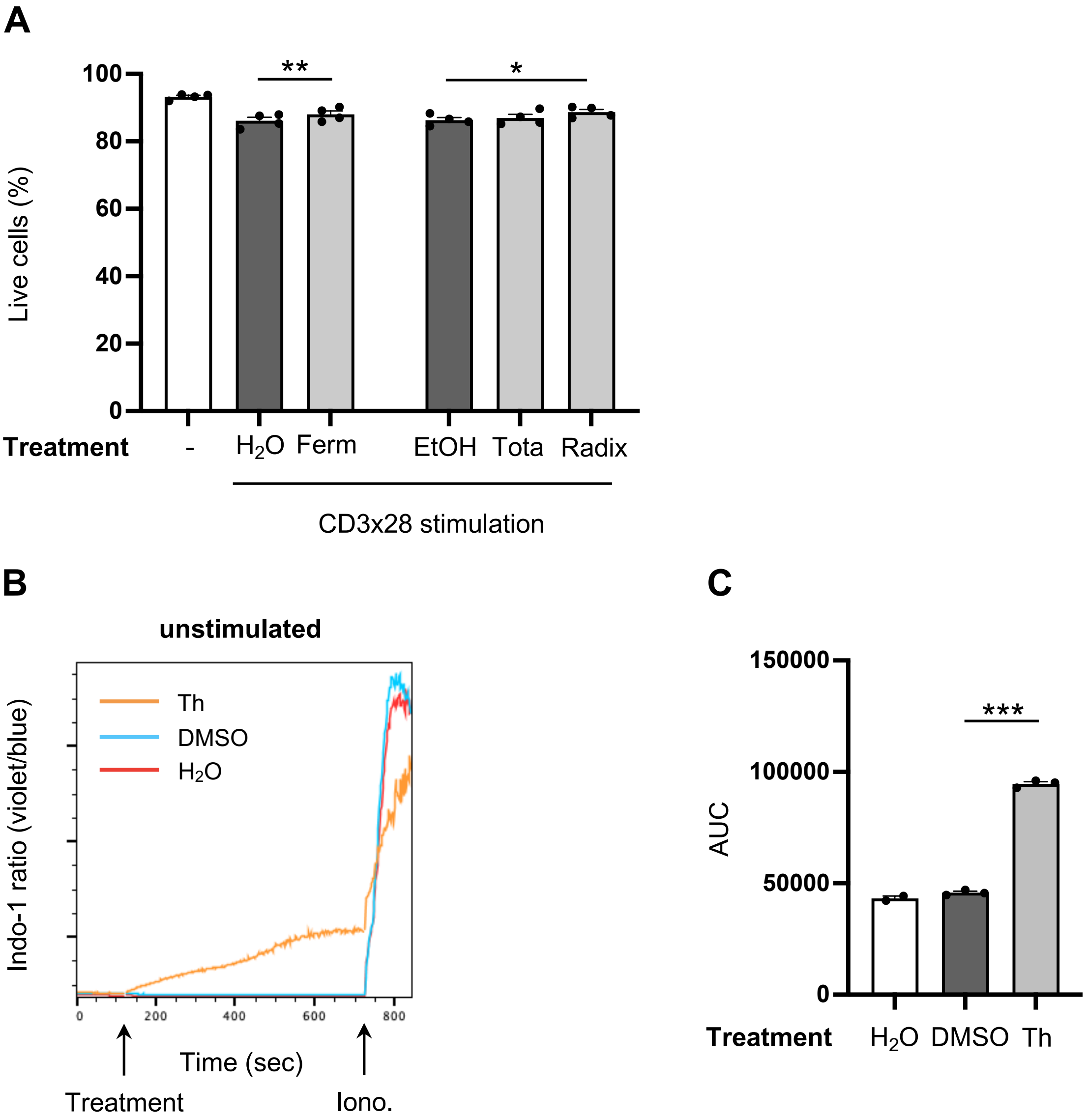

Suppl. Figure 8.

(**A**) **Jurkat cell viability after treatment with Arnica extracts.** Jurkat E6.1 cells were left unstimulated (white bar) or pretreated with Arnica extract (light grey bars) or vehicle (dark grey bars) for 1 h and subsequently activated with anti-CD3/CD28 antibodies for 6 h. Cell viability was quantified by 7-AAD exclusion in four independent experiments.

(**B**, **C**) **Intracellular calcium levels after treatment of PBTs with thymol.** PBTs were labeled with Indo-1 and left unstimulated. Changes in intracellular calcium levels upon addition of thymol or DMSO were assessed by flow cytometry. (**B**) Representative kinetics of intracellular calcium levels upon thymol treatment of unstimulated PBTs compared to solvent control sample. (**C**) Statistical evaluation of changes in intracellular calcium levels upon addition of thymol (light grey bar) or DMSO (dark grey bar) (three independent experiments). Each data point represents an individual T cell donor. Data are expressed as mean ± SEM. $^{*}p \leq 0.05$; $^{**}p \leq 0.01$; $^{***}p \leq 0.001$.
