# Supplementary material for: Distinct Arnica montana L. extracts modulate human T cell activation in different ways via differential inhibition of NFκB and NFAT pathways
Source: Front Immunol. 2025 Oct 15;16:1655212. doi: 10.3389/fimmu.2025.1655212 (PMC12568507; doi:10.3389/fimmu.2025.1655212)
Supplement: Supplementary file 1 [file DataSheet1.zip › BerschneiderK_ArnicaExtracts_SupplementaryTable-S1.pdf]

**Table S1: LC-MS/MS results (ESI-) of the examined commercial *Arnica montana* extracts.** Ferm: aqu. total ferm, Tota: ethanolic total extract, Radix: ethanolic root tincture. *: $t_R$ is based on the MS data. Delay time 0.06–0.09 min; $t_0$ was not subtracted (1.85 min in PDA). **: bold x marks the dominant compound ion of the corresponding peak; (x): shifted and spread over a wider area; N/A: not available; nl: neutral loss.

| Peak Nr. | Retention time [min]* | m/z | | Tentative identification | Occurrence in *A. montana* extracts | | |
|---|---|---|---|---|---|---|---|
| | | Precursor ion ** | Fragmentation pattern MS2 (relative abundance [%]) | | Ferm | Tota | Radix |
| 1 | 7.9–10.8 | 352.98 | 179.15 (8), 191.17(100) | Caffeoylquinic acid derivative (20, 21) | | x | |
| 2 | 14.0–14.3 | 443.18 | 160.78 (13), 178.75 (27), 381.10 (100), 399.13 (73), 425.13 (32) | N/A | x | x | |
| | | 489.05 | 425.15 (5), 443.10 (100), 471.04 (12) | | **x** | **x** | |
| 3 | 15.0–16.2 | 197.14 | 107.36 (5), 133.37 (6), 149.19 (23), 161.04 (100), 178.87 (7) | Trihydroxythymol (22) | x | x | (x) |
| 4 | 18.2–19.9 | 353.14 | 191.2179.39 (8), 191.21 (100) | Caffeoylquinic acid derivative (20, 21) | | | x |
| | | 707.04 | 295.15 (14), 334.40 (9), 352.77 (100), 470.36 (19) | Caffeoylquinic acid derivative dimer | | | **x** |
| 5 | 21.0–21.3 | 489.13 | 425.08 (100) | N/A | x | | |
| 6 | 24.1–24.4 | 165.11 | 146.97 (100) | N/A | x | | |
| 7 | 25.1–25.4 | 473.16 | 427.07 (100) | N/A | x | | |
| 8 | 25.1–25.8 | 463.27 | 160.93 (20), 232.70 (5), 331.09 (100) | N/A | | | x |
| | | 509.08 | 463.21 (100) | | | | **x** |
| 9 | 27.2–28.2 | 515.15 | 179.10 (24), 335.06 (31), 353.08 (100) | Dicaffeoylquinic acid derivative (21) | | x | x |
| 10 | 29.9–32.3 | 471.10 | 425.12 (100) | N/A | x | x | |
| | | 525.08 | 455.77 (3), 478.28 (5), 481.30 (100) | | | **x** | x |
| | | 611.27 | 251.09 (41), 431.14 (100) | | | x | |
| 11 | 31.9–32.4 | 525.11 | 481.30 (100) | N/A | x | | **x** |
| | | 593.09 | 481.26 (89), 503.05 (100), 547.08 (23) | | | | x |
| 12 | 35.8–36.6 | 449.18 | 209.10 (3), 225.08 (1), 251.12 (8), 269.10 (100), 287.13 (3), 431.19 (3) | N/A | x | x | |
| 13 | 37.4–38.7 | 373.11 | 167.03 (4), 329.11 (100) | N/A | x | x | x |
| | | 535.09 | 373.14 (100) | | | | **x** |
| 14 | 38.8–39.4 | 477.06 | 272.90 (1), 301.13 (100), 409.00 (1), 430.95 (2) | Quercetin-glucuronide / Quercitin-*O*-rhamnoside isomer (23, 24) | | x | |
| 15 | 39.7–40.3 | 515.22 | 174.24 (5), 179.01 (5), 203.20 (21), 255.34 (7), 299.11 (5), 317.15 (15), 348.65 (5) 352.93 (100), 441.90 (9), 469.06 (11), 470.82 (4), 498.60 (6) | Dicaffeoylquinic acid derivative (21) | | x | |
| | | 605.30 | 481.18 (4), 561.34 (100) | N/A | | **x** | |

| Peak Nr. | Retention time [min]* | m/z | | Tentative identification | Occurrence in *A. montana* extracts | | |
|---|---|---|---|---|---|---|---|
| | | Precursor ion ** | Fragmentation pattern MS2 (relative abundance [%]) | | Ferm | Tota | Radix |
| 16 | 41.2–41.5 | 287.22 | 191.18 (2), 209.06 (7), 225.16 (4), 227.10 (41), 251.08 (10), 269.09 (100) | N/A | x | | |
| 17 | 40.2–41.6 | 515.15 | 179.07 (2), 191.31 (3), 298.95 (5), 317.01 (6), 335.05 (8), 353.02 (100) | Dicaffeoylquinic acid derivative (21) | | x | x |
| 18 | 41.6–43.0 | 515.05 | 191.13 (2), 335.06 (4), 353.01 (100) | Dicaffeoylquinic acid derivative (21) | | x | x |
| 19 | 42.2–44.4 | 515.10 | 191.31 (4), 335.26 (5), 353.02 (100) | Dicaffeoylquinic acid derivative (21) | | x | x |
| 20 | 44.8–45.4 | 231.25 | 151.19 (35), 169.26 (29), 194.99 (4), 213.11 (100) | N/A | x | | |
| 21 | 46.3–46.9 | 217.28 | 141.29 (3) 171.21 (100), 199.14 (8) | N/A | x | | |
| 22 | 47.0–47.7 | 515.14 | 179.19 (4), 203.21 (15), 255.30 (6), 299.14 (16), 317.17 (9), 353.07 (100) | Dicaffeoylquinic acid derivative (21) | | | x |
| 23 | 48.7–49.8 | 677.18 | 335.13 (7), 353.09 (15), 497.15 (100), 515.05 (35) | Tricaffeoylquinic acid derivative (20, 26, 26) | | | x |
| 24 | 49.7–50.2 | 447.28 | 148.79 (3), 158.82 (2), 160.90 (19), 315.07 (100) | N/A | x | | |
| | | 493.08 | 447.23 (100) | | **x** | | |
| 25 | 50.1–51.0 | 471.07 | 245.01 (2), 381.17 (8), 407.12 (2), 422.99 (3), 425.03 (100) | N/A | | x | |
| | | 517.12 | 425.17 (8), 471.02 (100) | | | **x** | |
| 26 | 50.2–51.0 | 493.18 | 447.23 (100) | N/A | | | **x** |
| | | 677.18 | 335.17 (8), 353.12 (16), 469.12 (3), 497.15 (100), 515.04 (35) | Tricaffeoylquinic acid derivative (20, 26, 26) | | | x |
| 27 | 51.6–52.5 | 677.19 | 335.27 (8), 353.19 (19), 469.28 (3), 497.17 (100), 515.05 (35) | Tricaffeoylquinic acid derivative (20, 26, 26) | | | x |
| 28 | 52.8–53.9 | 207.20 | 135.46 (4), 161.45 (6), 179.30 (53), 207.20 (100) | Ethyl caffeate (22, 27) | | x | |
| 29 | 53.0–55.0 | 609.16 | 379.38 (38), 389.23 (27), 413.97 (78), 447.23 (78), 450.67 (43), 477.42 (49), 485.44 (74), 507.15 (49), 519.14 (59), 522.92 (100), 535.38 (23), 551.22 (29), 565.09 (36), 591.49 (33) | N/A | | | x |
| 30 | 53.7–54.5 | 609.11 | 565.47 (100) | N/A | x | | |
| 31 | 53.9–54.6 | 609.17 | 491.24 (100) | N/A | | x | |

| Peak Nr. | Retention time [min]* | m/z | | Tentative identification | Occurrence in *A. montana* extracts | | |
|---|---|---|---|---|---|---|---|
| | | Precursor ion ** | Fragmentation pattern MS2 (relative abundance [%]) | | Ferm | Tota | Radix |
| 32 | 55.4–57.1 | 421.16 | 173.06 (11), 179.10 (37), 203.13 (20), 258.95 (100), 299.02 (20), 335.13 (82), 353.03 (70) | N/A | | | x |
| | | 583.15 | 259.13 (7), 403.28 (7), 420.98 (100) | | | | **x** |
| 33 | 57.1–59.1 | 421.17 | 172.96 (17), 179.09 (24), 203.16 (14), 259.08 (54), 298.94 (22), 335.06 (92), 353.04 (100) | N/A | | | x |
| | | 583.15 | 255.19 (9), 299.05 (29), 317.06 (12), 403.09 (10), 421.04 (100) | | | | **x** |
| 34 | 56.8–59.8 | 266.90 | 127.39 (2), 149.05 (2), 161.01 (100), 178.69 (76) | N/A | x | x | |
| | | 689.27 | 565.20 (4), 645.37 (100) | | **x** | **x** | x |
| 35 | 59.1–62.1 | 609.16 | 563.10 (98), 565.16 (100), 590.58 (62) | N/A | | **x** | |
| | | 609.10 | 381.06 (62), 485.09 (100), 565.18 (31), 591.31 (64) | | | | x |
| | | 677.17 | 299.18 (3), 317.15 (3), 353.26 (4), 469.34 (19), 497.15 (63), 515.08 (100) | Tricaffeoylquinic acid derivative (20, 26, 26) | | x | **x** |
| 36 | 63.5–65.3 | 565.39 | 301.21 (7), 463.33 (100), 481.28 (13) | N/A | | x | x |
| | | 611.13 | 436.05 (24), 459.91 (100), 566.81 (13), 593.51 (67) | N/A | | **x** | |
| 37 | 65.3–65.7 | 327.28 | 155.11 (5), 171.28 (40), 201.47 (6), 209.35 (18), 211.24 (41), 221.24 (31), 229.27 (93), 239.27 (37), 247.34 (8), 291.29 (100), 309.26 (45) | N/A | x | x | |
| 38 | 65.6–66.0 | 609.21 | 452.95 (40), 565.27 (100) | N/A | | x | |
| | | 609.21 | 447.36 (51), 489.21 (100), 519.04 (32) | Lucenin-2 (25) | | | x |
| 39 | 66.0–66.31 | 609.21 | 179.07 (20), 255.11 (24), 447.11 (100), 497.18 (40), 500.85 (16), 515.84 (21), 565.30 (37) | N/A | | | **x** |
| | | 677.13 | 265.09 (3), 317.10 (2), 469.21 (16), 497.05 (78), 515.12 (100) | Tricaffeoylquinic acid derivative (20, 26, 26) | | | x |
| 40 | 66.1–66.6 | 299.18 | 284.17 (100) | Hispidulin (23) | | x | |
| 41 | 67.2–68.5 | 281.00 | 161.04 (100), 178.76 (80), 213.09 (3) | N/A | | x | |
| | | 645.53 | 241.30 (3), 463.50 (8), 543.41 (100), 565.30 (5) | N/A | | x | x |
| | | 785.58 | 545.31 (14), 639.37 (13), 665.30 (100) | N/A | | x | |
| 42 | 68.7–74.1 | 745.18 | 299.16 (3), 403.27 (6), 421.27 (23), 537.27 (76), 565.12 (89), 583.01 (100) | N/A | | | x |
| 43 | 68.7–69.7 | 329.30 | 171.45 (9), 211.34 (40), 229.29 (100), 293.22 (15), 311.32 (44) | N/A | x | x | |
| 44 | 71.0–71.3 | 785.45 | 545.36 (15), 639.39 (13), 665.30 (100) | N/A | | x | |

| Peak Nr. | Retention time [min]* | m/z | | Tentative identification | Occurrence in *A. montana* extracts | | |
|---|---|---|---|---|---|---|---|
| | | Precursor ion ** | Fragmentation pattern MS2 (relative abundance [%]) | | Ferm | Tota | Radix |
| 45 | 72.6–72.9 | 329.28 | 181.13 (4), 199.40 (6), 211.23 (17), 293.22 (22), 311.24 (100) | N/A | x | | |
| 46 | 73.0–73.2 | 477.23 | 299.08 (94), 311.14 (79), 339.28 (6), 369.15 (100), 459.13 (55) | N/A | | x | |
| 47 | 74.6–75.0 | 693.13 | 310.87 (19), 339.33 (19), 501.22 (29), 513.30 (51), 517.96 (21), 531.05 (100), 604.19 (8), 646.27 (15), 657.08 (16), 675.14 (14) | N/A | | x | |
| | | 693.18 | 85.36 (9), 531.09 (41), 611.91 (11), 624.69 (12), 649.29 (100), 675.35 (13) | N/A | | | x |
| 48 | 77.0–78.6 | 713.43 | 477.21 (11), 491.23 (14), 561.24 (14), 681.28 (32), 698.19 (36), 713.38 (100) | N/A | | x | |
| | | 713.43 | 478.20 (10), 561.21 (17), 681.30 (35), 698.22 (34), 713.34 (100) | | | | x |
| 49 | 79.0–79.8 | 649.47 | 202.86 (2), 301.17 (3), 463.35 (2), 547.31 (100), 565.32 (42) | N/A | | | **x** |
| | | 694.98 | 528.61 (100), 536.33 (59) | N/A | | | x |
| 50 | 81.0–81.9 | 649.42 | 300.93 (3), 463.28 (2), 547.31 (100), 565.31 (28) | N/A | | | x |
| 51 | 82.5–82.8 | 491.17 | 297.20 (8), 313.14 (100), 383.17 (58), 473.15 (28) | N/A | | x | |
| 52 | 83.2–84.0 | 337.01 | 161.06 (100), 219.05 (100), 248.87 (57) | N/A | | | x |
| 53 | 84.4–84.7 | 601.49 | 187.20 (4), 261.16 (9), 431.20 (100), 583.42 (7) | N/A | | x | |
| 54 | 87.0–88.0 | 351.01 | 101.08 (3), 133.16 (2), 161.06 (100), 219.06 (45), 233.06 (53), 248.86 (26), 262.88 (34) | N/A | | | x |
| 55 | 88.6–89.1 | 489.32 | 297.20 (6), 429.28 (6), 443.13 (10), 474.12 (100) | N/A | | x | |
| 56 | 89.6–90.1 | 476.35 | 279.3196.16 (59), 214.18 (39), 279.30 (100), 407.05 (8), 415.24 (19), 431.44 (6), 458.00 (5) | N/A | | | x |
| 57 | 90.3–91.2 | 649.19 | 410.46 (26), 474.20 (28), 520.64 (55), 569.25 (25), 602.98 (100) | N/A | | | x |
| 58 | 91.3–91.9 | 564.27 | 504.23 (100) | N/A | | | x |
| 59 | 92.5–93.5 | 564.31 | 504.25 (100) | N/A | | | x |
| 60 | 93.7–95.0 | 595.39 | 233.03 (3), 241.15 (19), 279.30 (2), 315.14 (63), 333.08 (3), 415.18 (100) | N/A | | | x |
| 61 | 96.5–96.8 | 416.14 | nl | N/A | x | | |
| 62 | 95.0–96.6 | 595.42 | 241.19 (10), 315.13 (28), 415.16 (100) | N/A | | | x |
| 63 | 96.8–98.1 | 540.28 | 480.29 (100) | N/A | | | x |
| 64 | 98.5–102.5 | 571.41 | 241.19 (11), 315.13 (37), 391.16 (100) | N/A | | | x |
| 65 | 105.8–108.2 | 433.34 | 153.12 (100), 171.03 (3) | N/A | | | x |
| 66 | 107.8–108.6 | 297.31 | 251.44 (1), 297.28 (100) | N/A | | x | |

| Peak Nr. | Retention time [min]* | m/z | | Tentative identification | Occurrence in *A. montana* extracts | | |
|---|---|---|---|---|---|---|---|
| | | Precursor ion ** | Fragmentation pattern MS2 (relative abundance [%]) | | Ferm | Tota | Radix |
| 67 | 109.6–111.2 | 529.27 | 161.04 (76), 219.03 (45), 248.89 (32), 279.34 (5), 411.19 (51), 441.05 (100), 461.40 (6), 482.84 (4), 493.63 (4) | N/A | | | x |
| | | 730.59 | 271.24 (16), 326.23 (100), 550.27 (47), 568.46 (42) | N/A | | | x |
| | | 776.31 | 309.03 (25), 321.28 (28), 334.92 (24), 382.50 (54), 399.26 (44), 481.32 (26), 622.03 (24), 640.71 (41), 661.22 (41), 707.19 (73), 714.59 (26), 717.63 (26), 729.53 (100), 739.38 (27), 758.30 (50) | N/A | | | **x** |
| 68 | 111.8–112.2 | 379.35 | 117.12 (3), 335.19 (9), 343.29 (34), 361.22 (100), 375.23 (6) | N/A | | | x |
| 69 | 112.5–113.9 | 776.32 | 422.42 (42), 443.64 (35), 459.14 (31), 485.85 (32), 493.43 (58), 534.36 (38), 538.05 (35), 635.53 (43), 707.36 (54), 715.31 (48), 729.02 (100), 748.22 (34), 758.71 (63) | N/A | | | x |
| 70 | 115.2–115.7 | 339.25 | 297.27 (100) | N/A | | **x** | |
| | | 701.53 | 297.33 (6), 339.16 (13), 421.11 (100) | | | x | |
