# Supplementary material for: Distinct Arnica montana L. extracts modulate human T cell activation in different ways via differential inhibition of NFκB and NFAT pathways
Source: Front Immunol. 2025 Oct 15;16:1655212. doi: 10.3389/fimmu.2025.1655212 (PMC12568507; doi:10.3389/fimmu.2025.1655212)
Supplement: Supplementary file 1 [file DataSheet1.zip › BerschneiderK_ArnicaExtracts_SupplementaryTable-S2.pdf]

Table S2: Summary of significantly up-or downregulated genes upon treatment with Arnica preparations.

For each significantly regulated gene ($p \leq 0.05$), the ratio (log2) of differential expression upon treatment with the different test drugs compared to their respective solvent control is indicated in numbers.

Gene	ratio (log2)				
	Ferm	Tota	Radix	Thymol	Helenalin
ABCB1		0.42			0.60
AHR			0.48	-0.42	
ARG2				1.02	
ARHGDIB				0.91	
ATG16L1				0.26	
BATF		-0.73		-1.64	-1.27
BATF3		-0.36	-0.50	-1.71	-0.64
BAX	0.22	0.20	0.17	0.25	0.21
BCAP31		0.33			
BCL10	0.15	0.28	0.51	0.55	0.30
BCL2				0.92	
BCL2L11		-0.47	-0.51	-1.27	-0.71
BCL6			0.59	0.79	-0.39
BTK	-0.68			0.65	
BTLA					-0.80
C8A				0.63	
CASP2				0.61	
CASP8				0.67	
CCL20		-0.36		-0.92	-1.04
CCND3				0.95	
CCR7				2.18	
CD27				0.94	
CD274			-0.81	-1.24	-0.41
CD28	0.15			1.44	
CD3D				0.51	
CD3E				0.42	
CD3EAP				-0.54	-0.39
CD40LG				-1.32	
CD45RA				0.98	
CD45RB				0.78	
CD48		-0.23		0.26	-0.32
CD5				2.14	
CD59		0.44	0.38		0.32
CD6				1.28	
CD7				0.64	-0.58
CD82		-0.42		-0.67	-0.72
CD96			0.30	0.70	
CD97					-0.49
CEBPB		0.31	0.79	0.72	0.31
CHUK				-0.33	
CISH	-0.29	-0.70	-0.87	-2.20	-0.86
CRADD				0.78	
CSF1				-0.65	
CSF2		-0.83	-0.82	-3.04	-1.36
CTLA4_all		-0.58		0.69	-0.49
CTLA4-TM		-0.55		0.55	-0.62
CTNNB1		0.14	0.14		0.16
CUL9				0.57	
CXCR4				2.01	
DUSP4		-0.83			-1.12
EGR2					-0.37
FKBP5			-0.29		
FOXP3	-0.44			0.61	
FYN				0.34	-0.24
GBP1				-1.34	-0.28
GFI1	-0.39	-0.60		-0.49	-0.92
GZMB				-1.99	
ICAM1				-1.27	
ICAM2				0.39	
ICAM3					0.23
ICOS		-0.46			-0.56
IFI16					-0.19
IFITM1		-0.52	-0.95	-0.65	
IFNAR1				0.78	
IFNAR2			0.41	1.18	
IFNG		-0.81		-2.41	-1.31
IFNGR1		0.19			
IL10	-0.79	-1.72	-1.41	-1.86	-1.83
IL16				2.62	
IL17A	-0.45	-1.05	-0.82	-1.60	-1.49
IL17F				-1.43	-1.19
IL18R1				-0.83	
IL18RAP		-0.68		-0.88	-0.81
IL1R1			0.49		-0.59
IL1RL1		-0.77			
IL2	-0.75	-1.28		-2.51	-1.95
IL21		-1.11	-0.93	-2.12	-1.39
IL21R					-0.53
IL22		-1.02	-0.64	-2.92	-1.53
IL23A				-0.71	
IL23R			0.31		
IL2RA				-1.25	-0.59
IL3				-3.55	-1.66
IL4				-1.34	
IL4R					-0.62
IL7R			0.66	0.65	
ILF3		-0.35			-0.38
IRAK1					-0.30

Gene	ratio (log2)				
	Ferm	Tota	Radix	Thymol	Helenalin
IRF1				-0.46	
IRF4		-0.39			-0.61
IRF8	-0.60	-0.88	-1.01	-1.77	-1.16
ITGA5				1.41	
ITGA6		0.56		-0.55	
ITGAE				0.67	
JAK1			0.34		
JAK2	-0.50	-0.36		-1.27	-0.54
JAK3		-0.56			-0.53
KIR_Activating_Subgroup_1				0.80	
KIR_Inhibiting_Subgroup_2				0.61	
KLRB1				0.69	
KLRC1				0.79	
KLRC2				0.67	
KLRC4			0.46		
KLRK1				1.00	
LAMP3	-0.28		-0.47	-1.47	-0.54
LCK			0.19	0.88	
LCP2		-0.47	-0.29	-0.35	-0.57
LEF1				0.94	
LIF		-0.82	-0.82	-1.60	-0.83
LILRA4				0.75	0.52
LTA	-0.34	-0.71	-0.98	-2.66	-1.13
LTB4R2	-0.35				
MAF		-0.40			
MALT1				-0.61	
MAP4K2				0.98	
MAPK14				0.64	
MAPKAPK2		-0.34			
MCL1				0.42	
MIF					-0.54
MYD88		-0.11	-0.49		
NFATC3				0.43	
NFIL3				-0.97	
NFKB1					-0.38
NFKBIA				-0.61	
NOTCH1	-0.38	-0.53		-0.70	-0.55
NOTCH2				-0.54	
PDCD1				0.33	
PLAU				-0.87	
POU2F2					-0.60
PRKCD				-0.43	
PSMB7				-0.28	
PSMB8			-0.34	-0.40	-0.35
PSMB10					-0.37
PSMC2		0.20		0.23	0.13
PSMD7			0.23		
PTGER4				-0.28	-0.40
PTPN2				-0.33	-0.28
PTPN22				0.67	
PTPN6		-0.39	-0.43	-0.48	-0.45
PTPRC_all				0.78	
RELB				-0.37	-0.30
RORC		-0.38		0.70	-0.35
SELL				-0.50	
SKI			-0.25	-0.33	0.20
SLAMF6				0.85	
SLC2A1			0.41		
SOCS1			-0.56	-1.22	
SOCS3			-0.89	-1.51	
STAT5A	-0.31	-0.47		-0.43	-0.71
STAT5B		-0.29		0.64	-0.40
STAT6					-0.60
TAGAP		-0.38		0.43	-0.47
TAP1			-0.40	-0.97	
TAP2				-0.38	-0.24
TAPBP	-0.18			-0.51	-0.52
TBX21				-1.08	
TCF7				0.78	
TFRC				-1.21	
TGFB1		-0.19	-0.28	-0.58	-0.28
TNF		-0.48	-0.53	-1.39	-0.71
TNFAIP3	-0.45	-0.37			-0.61
TNFRSF13C					-0.45
TNFRSF14				0.55	
TNFRSF4		-1.02		-1.04	-1.36
TNFRSF9	-0.40	-0.71		-0.56	-1.07
TNFSF10				-1.21	
TNFSF11				-0.82	
TNFSF8		-0.84		-1.91	-1.23
TOLLIP				0.23	
TP53				0.48	
TRAF2					-0.20
TRAF4				-0.38	-0.39
TRAF5		-0.36		0.28	-0.38
TYK2				0.35	
XCL1				-1.22	

downregulated genes

upregulated genes
